# Clinical Decision Support Systems for Drug Allergy Checking: Systematic Review

**DOI:** 10.2196/jmir.8206

**Published:** 2018-09-07

**Authors:** Laura Légat, Sven Van Laere, Marc Nyssen, Stephane Steurbaut, Alain G Dupont, Pieter Cornu

**Affiliations:** ^1^ Research Group Clinical Pharmacology and Clinical Pharmacy Centre for Pharmaceutical Research Vrije Universiteit Brussel Brussels Belgium; ^2^ Research Group of Biostatistics and Medical Informatics Department of Public Health Vrije Universiteit Brussel Brussels Belgium

**Keywords:** alert, clinical decision support systems, computerized physician order entry, drug allergy, patient safety

## Abstract

**Background:**

Worldwide, the burden of allergies—in particular, drug allergies—is growing. In the process of prescribing, dispensing, or administering a drug, a medication error may occur and can have adverse consequences; for example, a drug may be given to a patient with a documented allergy to that particular drug. Computerized physician order entry (CPOE) systems with built-in clinical decision support systems (CDSS) have the potential to prevent such medication errors and adverse events.

**Objective:**

The aim of this review is to provide a comprehensive overview regarding all aspects of CDSS for drug allergy, including documenting, coding, rule bases, alerts and alert fatigue, and outcome evaluation.

**Methods:**

The Preferred Reporting Items for Systematic Reviews and Meta-Analyses (PRISMA) guidelines were followed as much as possible and searches were conducted in 5 databases using CPOE, CDSS, alerts, and allergic or allergy as keywords. Bias could not be evaluated according to PRISMA guidelines due to the heterogeneity of study types included in the review.

**Results:**

Of the 3160 articles considered, 60 met the inclusion criteria. A further 9 articles were added based on expert opinion, resulting in a total of 69 articles. An interrater agreement of 90.9% with a reliability *Κ*=.787 (95% CI 0.686-0.888) was reached. Large heterogeneity across study objectives, study designs, study populations, and reported results was found. Several key findings were identified. Evidence of the usefulness of clinical decision support for drug allergies has been documented. Nevertheless, there are some important problems associated with their use. Accurate and structured documenting of information on drug allergies in electronic health records (EHRs) is difficult, as it is often not clear to healthcare providers how and where to document drug allergies. Besides the underreporting of drug allergies, outdated or inaccurate drug allergy information in EHRs poses an important problem. Research on the use of coding terminologies for documenting drug allergies is sparse. There is no generally accepted standard terminology for structured documentation of allergy information. The final key finding is the consistently reported low specificity of drug allergy alerts. Current systems have high alert override rates of up to 90%, leading to alert fatigue. Important challenges remain for increasing the specificity of drug allergy alerts. We found only one study specifically reporting outcomes related to CDSS for drug allergies. It showed that adverse drug events resulting from overridden drug allergy alerts do not occur frequently.

**Conclusions:**

Accurate and comprehensive recording of drug allergies is required for good use of CDSS for drug allergy screening. We found considerable variation in the way drug allergy are recorded in EHRs. It remains difficult to reduce drug allergy alert overload while maintaining patient safety as the highest priority. Future research should focus on improving alert specificity, thereby reducing override rates and alert fatigue. Also, the effect on patient outcomes and cost-effectiveness should be evaluated.

## Introduction

Worldwide, the burden of allergies is growing—in particular, drug allergies (DAs) are becoming increasingly common [1]. DAs can be categorized as abnormal immunoglobulin E-mediated reactions (eg, anaphylaxis) or delayed, nonimmunoglobulin E-mediated reactions, which are generally less severe (eg, intolerances) [2].

DAs are perceived as an important problem. In a study conducted by the European Network on Drug Allergy and the EAACI Drug Allergy Interest group, 10% of parents reported that their child was allergic to a drug [3]. A study in a tertiary care academic medical center in Chicago reported a DA prevalence of 25% in the general adult population [4]. Looking at the clinical investigations of suspected reactions, the results demonstrate that these numbers are overvalued [3]. In a general hospital in Singapore, the estimated incidence of DAs was 4.20 per 1000 hospitalizations (95% CI 2.93-5.46) and the estimated mortality attributable to DA was 0.09 per 1000 hospitalizations (95% CI 0.06-0.12) [5]. A study in a university hospital in Korea reported an estimated incidence of drug hypersensitivity reactions of 1.8 per 1000 hospital admissions [6].

In the process of prescribing, dispensing, or administering a drug, a medication error can occur and may have adverse consequences, for example, when a drug is given to a patient with a documented DA to this particular drug [7]. Only a minority (0.25%) of these medication errors result in an adverse drug event (ADE), but allergic reactions represent an important cause of preventable ADEs caused by medication errors [8,9]. It was estimated that 12.1% of all medication errors with the potential for an ADE arise from incomplete or incorrect allergy documentation [10].

Bates et al [11] and Classen et al [12] estimated that each ADE resulted in a prolonged length of hospital stay of 2.2 and 1.7 days, respectively. Looking more specifically at penicillin allergy, Macy et al [13] demonstrated that in the Kaiser Foundation Hospitals in Southern California, 0.59 additional hospital days (95% CI 0.47-0.71) per hospitalization resulted in an extra cost of US $1252.90 in 2012.

CPOE systems with built-in CDS have the potential to prevent such medication errors and consequent ADEs [14-16]. When a prescription poses a threat to the patient, the clinical decision support system (CDSS) warns the user by providing an alert message. However, it is well known that current CDSS for DA checking are impaired by alert fatigue caused by low alert specificity [17-19].

Several systematic reviews have been conducted to evaluate CPOE and CDSS in general or in specific domains of clinical care such as pediatrics [14-16,19-28]. To the best of our knowledge, no systematic review has been conducted focusing specifically on CDSS for DA. In this systematic review, we aimed to provide a comprehensive overview of all aspects of CDSS for DA including documenting, coding, rule bases, alerts and alert fatigue, and outcome evaluation.

## Methods

### Search Strategy

**Figure 1:**
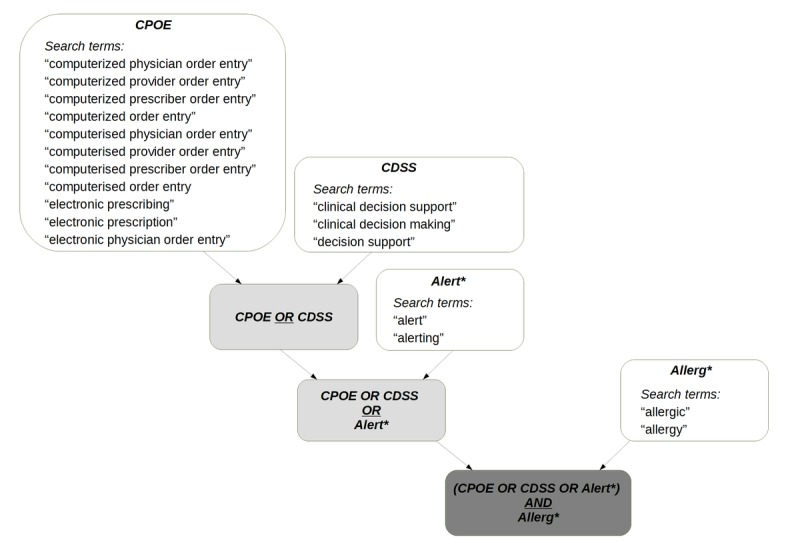
Search strategy used for navigating the 5 libraries (Cumulative Index to Nursing and Allied Health Literature, Cochrane, Embase, Ovid, and MEDLINE). The matching search terms are listed in the lower part of the figure. CDSS: clinical decision support system; CPOE: Computerized Physician Order Entry.

A systematic literature review was performed following the Preferred Reporting Items for Systematic Reviews and Meta-Analyses (PRISMA) guidelines for systematic reviews and meta-analyses [29] as much as possible. Bias could not be evaluated according to the PRISMA guidelines due to the heterogeneity of study types included in the review. Here, we focused on searching for articles related to CDSS and associated alerts in the domain of DAs. We performed searches in the bibliographic libraries of Cumulative Index to Nursing and Allied Health Literature (CINAHL), Cochrane Library, Embase, Ovid, and PubMed from database inception up to February 2016. The search strategy for the 5 databases is provided in Multimedia Appendix 1. Because the aim of the review was to provide a broad overview of all aspects of DA-related CDSS, reviews and conference proceedings were also included. Only English language papers were included. Additional publications of interest that included information relevant to this review were included based on expert opinion. Our search strategy is presented in Figure 1. The terms “Computerized Physician Order Entry” (CPOE) and “Clinical Decision Support System” (CDSS) were combined with the term “alert.” These terms were combined with the term “allergic” or “allergy” to limit the scope to the allergy field.

### Study Selection

**Figure 2:**
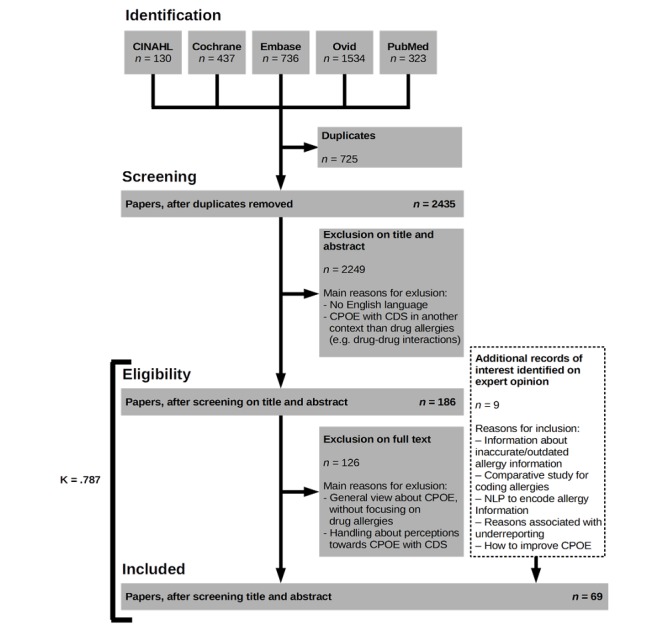
Search strategy (Preferred Reporting Items for Systematic Reviews and Meta-Analyses), with reasons for exclusion and inclusion of articles added based on expert opinion. CDS: clinical decision support; CINAHL: Cumulative Index to Nursing and Allied Health Literature; CPOE: computerized physician order entry systems; NLP: natural language processing.

The titles and abstracts of identified articles were independently screened by two researchers (LL and SVL) to assess inclusion in the full review (Figure 2). If one or both reviewers selected the paper for further evaluation, we included the article for full assessment. Articles were included for analysis if the study involved at least one of the following: (1) prevalence of allergy alerts; (2) coding or documenting of DA information; (3) implementation of a CDSS for DA; (4) perceptions of care providers on CDS for DAs; or (5) alert acceptance and interface design in the domain of allergies. Disagreements were discussed with a third reviewer (PC) until consensus was reached.

### Data Extraction

From each article included, the two researchers (LL and SVL) extracted predefined information including the author names, year of publication, main topic of the paper, aim of the study, study design, number of subjects (care providers, alerts, etc), and key findings. The third reviewer (PC) evaluated the extracted data, and disagreements were resolved by consensus.

Assessment of risk of bias was not conducted because the heterogeneity in the quantitative and qualitative study designs, reviews, and reports did not allow for a comprehensive and consistent evaluation of bias.

## Results

### Study Selection and Reviewer Agreement

We started the study with 3160 articles from 5 different literary sources (Figure 2). After the removal of duplicates (725 duplicates), 2435 articles remained for title and abstract review. Eventually, 186 articles were included for full text review, of which 60 were included in this review. The interrater reliability between LL and SVL was calculated using Cohen’s kappa. An interrater agreement of 90.9% with a reliability of *Κ*=.787 (95% CI 0.686-0.888) was reached between the two reviewers. Additionally, 9 articles were added based on expert opinion, resulting in a total selection of 69 articles. Although these 9 articles were not retrieved by the search query, they reported on aspects relevant for this review, including information related to the coding of allergy information, reporting of DAs, and strategies for improving CPOE alerts. These articles were identified by PC from automatic weekly updates on PubMed (My NCBI). These weekly updates were based on separate queries with individual key words including “Computerized Physician Order Entry,” “Clinical Decision Support,” “Medication error,” and “Drug allergy.” PC collected and indexed relevant articles from this weekly list using reference manager software over the years. Articles included in the library of the reference software that dealt with relevant topics but were not discovered via the search queries were included.

### Study Characteristics

The names of the authors, year of publication, study design, aim of the study, and key findings are shown in Multimedia Appendix 2. Papers in Multimedia Appendix 2 are organized by topic [1,14,16-19,22,30-91]. Papers belonging to different topics are categorized in all corresponding topics. In Multimedia Appendix 3, a table with the number of subjects included in the study can be found. Most studies (56/69, 81%) were published after 2005. The 69 studies consisted of 28 observational studies (23 retrospective studies, 2 cross-sectional studies, 1 prospective time series analysis, 1 prospective study with interviews, and 1 cohort study), 13 review articles, 8 practice experiences, 6 before-after studies, 3 surveys, 3 controlled trials (1 randomized controlled trial, 1 randomized crossover study, and 1 nonrandomized controlled trial), 2 economic evaluations, 2 focus group studies, 1 scenario-based simulation study, 1 study describing a draft for an algorithm to classify information automatically, 1 study discussing a workshop, and 1 study describing a comparative study on standards in the DA field.

### Documenting the Presence or Absence of an Allergy

For a functional CDSS, accurate and consistent documentation of patient allergy information is necessary. Currently, there is no agreement about what needs to be recorded and how to do so [30]. In 1964, Mills addressed the importance of capturing information on allergies and drugs, proposing a new checklist that served as a guideline in hospitals [31]. A similar initiative was taken more recently by Burrell et al [32], who introduced a pharmacist-driven protocol in a hospital to improve the completeness of DA or intolerance documentation. A review of medical notes in a general district hospital demonstrated poor documentation practices where 97.4% (114/117) of drug allergy boxes were only partially completed and 2.6% (3/117) had nothing documented [33]. In another analysis comparing two oncology wards, one ward showed 100% consistency, while the other ward demonstrated drug charts with allergy entries in 82.4% of cases, of which only 68.8% corresponded to information in the medical notes [34]. Failure to accurately document DAs may lead to prescribing and administering medications that could be harmful to the patient. Besides accurate documentation, correct patient identification with linking of medication information with patient DA information, for example wristband barcoding, is required for real-time CDS [35].

Lopez-Gonzalez et al [38] reviewed factors for not reporting ADRs and found that the most prominent factor associated with underreporting was ignorance based on the fact that physicians often think that only severe ADRs need to be reported. Secondary factors, such as the hierarchical nature of hospital culture combined with stressful working conditions, also contributed to prescribing errors [41]. Moreover, at the time of documenting, there is often no clear distinction between a real allergy-related ADR and other minor reactions [37].

Inaccurate or outdated DA information can also be a factor that influences the functioning of a CPOE system with CDS. Rimawi et al [40] observed a small use case (150 patients) that was documented as intolerant to penicillin even though a negative penicillin skin test was observed. Of these, 36% (20/55) patients who revisited the medical center within the year were redocumented as having a penicillin allergy without proper indication.

Porter et al [39] demonstrated that without healthcare personnel re-asking and validating information, there is a significant risk of error at the decision step of ordering or prescribing medications. Additionally, side effects related to the drug’s primary pharmacological effect are sometimes misinterpreted and documented as DAs, resulting in inaccurate DA information.

The more complete and accurate DAs recorded in patients’ EHRs, the greater the potential of CPOE systems with CDS to improve patient safety and reduce medication-related costs [14].

### Coding

Information on how allergy data are structured or coded in EHRs is scarce. Slight et al [52] recently stated that the US government has not yet specified what standard terminologies should be used to structure allergy information.

A first approach is to enter information concerning DAs in free text. In order to use free text information for CDSS, natural language processing is applied taking the context into consideration [47]. Currently, this technique is not widely used because of the difficult nature of natural language processing.

A review of the available literature indicates that different coding systems are used for documenting DA information in EHRs, including International Classification of Diseases (ICD) [44,51], Systematized Nomenclature of Medicine, Clinical Terms (SNOMED CT) [49], RxNorm, and National Drug File-Reference Terminology (NDF-RT) [50]. Sometimes mapped coding schemes are used to add functionality. Benkhaial et al [44] used the Anatomical Therapeutic Chemical classification for mapping all drugs belonging to a specific ICD group. However, mapping might not always be this simple. Bernstein [45] recently indicated in a Danish use case that there is not yet a clear consensus regarding the alert information concept (eg, drug alerts) and how drug allergy or other allergy subtypes are linked to that concept.

Goss et al [48] performed a comparative study of the SNOMED CT, NDF-RT, Medication Dictionary for Regulatory Activities, unique ingredient identifier, and RxNorm standards for encoding allergy information. The qualitative part of their study demonstrated that SNOMED CT had the most desirable characteristics, including concept coverage, subset capabilities, and vocabulary structure. The quantitative part showed that RxNorm had the highest concept coverage to represent drug allergens, followed by unique ingredient identifier, SNOMED CT, NDF-RT, and Medication Dictionary for Regulatory Activities. SNOMED CT was the only coding system capable of representing unique concepts to encode ignorance of allergies.

The option to have an entry to indicate the ignorance of allergies is important for patient care because not documenting any DA information does not necessarily mean that there are no known DAs. Abookire et al [43] stated that “every hospitalized patient should have DAs entered by the admitting physician (this is a forced entry; 'no allergies' may be entered).”

### Rule Bases

In the literature, we observed underreporting of the rule bases used to support CDSS. Most CDSS for DA screening are knowledge-based systems supported by evidence-based rule databases. Some organizations use internally developed rule bases [54], while others use vendor-supplied rule bases [56].

We observed two types of CDSS in the literature: basic and complex. Basic CDSS provide alerts when a prescribed drug is listed in the patient’s DA list. In these systems, rules are implemented to screen for cross-reactivity within and between drug classes [55]. More complex CDSS use an inference mechanism to generate recommendations specific to a patient by integrating contextual information from the patient’s EHR (eg, previously tolerated administrations of the drug and results from DA tests) [55].

As reported by Kuperman et al [14], healthcare provider organizations ideally use a combination of vendor-supplied rule sets, which are developed by other organizations, and internally developed rules, which are derived from the literature and national and local consensus on what constitutes best practice. In any system, as medicine evolves and clinical knowledge grows, a timely review of the rule bases is warranted, for example using a Delphi approach to analyze what rules are useful [53].

### Alerts and Alert Fatigue

Alert fatigue has been defined as “declining physician responsiveness to a particular type of alert as the physician is repeatedly exposed to that alert over a period of time, gradually becoming ‘fatigued’ or desensitized to it” [60]. Alert fatigue caused by one type of alert may also lead to declined responsiveness to other types of CDS alerts (eg, drug-drug interaction, DDI, alerts). The state of the art in CDS for DA is such that alerts are not specific enough, resulting in high override rates [17,18]. Current systems, which generate an alert at the moment of prescribing, have very high override rates of over 90% [1,17]. The first concern regarding increasing DA alerting rates and overrides was raised by Abookire et al [43]. This problem has since been investigated in several other studies. Bryant et al [59] retrospectively analyzed physician responses to DDI and DA interaction alerts in two university hospitals and reported high override rates in all categories, ie, 92.87% (2280/2455 alerts) in general and 90.86% (1183/1302 alerts) for DAs. No significant difference in override rates was observed between hospitals or between physicians-in-training and residents. Topaz et al [80] demonstrated a significant increase in DA alert overrides from 83.3% in 2004 to 87.6% (*P*<.001) in 2013 in a retrospective longitudinal study of two large academic medical centers. Similarly, Lin et al [71] demonstrated an increase in drug allergy override rates from 72 overrides out of 105 alerts (68.6%) in 2001 to 341 overrides out of 420 alerts (81.2%) in 2006.

In a recent observational study, Slight et al [1] evaluated DA alerts generated over a 3-year period in a tertiary care teaching hospital and 36 primary care practices and found that in total, 81.10% (128,157/158,023) DA alerts in both settings were overridden. In a retrospective 5-month chart review study conducted by Genco et al [63], a similar override rate of 87.4% (153/175) DA alerts was observed and the overall override rate for all types of alerts was 93.51% (12,829/13,719).

When an alert is overridden, analyzing the override reasons can help to understand the specific context. Several studies have reported the reasons frequently given for DA alert nonadherence, including (1) “medication was previously tolerated”; (2) “known DA for which only monitoring is needed”; (3) “the benefit outweighs the risk”; and (4) “alert considered not clinically important” [66,67,79].

Besides evaluating override reasons, the underlying causes of high override rates should be investigated. Hsieh et al [37] cited two important causes. The first cause is highly inclusive drug class and drug cross-reactivity mapping, which generates a large number of DA alerts for drugs with only minor potential to cause an allergic reaction. Alert acceptance is more likely when the warning is infrequently encountered [68] and when the physician encounters an exact drug match instead of a drug class warning [77]. The second cause is difficulty maintaining accurate allergy lists because there may not be a clear distinction between immune-meditated allergies and nonimmune-mediated sensitivities, and there is no general consensus on whether both should be included in allergy lists [37]. The likelihood that alerts will be ignored is related to the low predictive value for allergic drug reactions and inaccurate alerts because of inconsistent information in medical records [37,69].

Strategies for improving alert specificity and acceptance have been proposed. Horsky et al [65] stated that the specificity and contextual relevance of alerts can be increased by periodically reviewing trigger rules, a thorough analysis of performance logs, and maintenance of accurate allergy, problem, and medication lists in EHRs. Additionally, Brodowy et al [58] demonstrated a reduction in DA alerts by simply eliminating alerts resulting from inactive ingredients.

The possibility of customizing CDSS to increase alert specificity and alert acceptance has also been reported [61]. CDSS, where the severity levels for drug or disease interactions can be modified by the physician to exclude alerts at a level considered not relevant, or the use of an on-demand approach that provides decision support only when a physician considers it relevant, could improve alert acceptance [78]. This may be related to the caregiver status of the person using CDSS. Knight et al [68] demonstrated, for example, that nurses are nearly twice as likely to accept an alert compared with a resident (odds ratio [OR] 1.92, 95% CI 1.44-2.57). The usability of the alerts can also be improved by applying human factors design principles [65,75]. For example, a tabular format for presenting multiple alerts and the grouping of similar information aid in making prescribing decisions [75]. Designing a workflow with minimum disruptions by only showing critical to high-severity alerts, as suggested by Shah et al [76], could also be effective.

### Outcomes

In the literature, we found limited results related to the outcomes of CDSS for DA. We did find studies that investigated the number of prescribing errors (PEs) and studies discussing patient outcomes for different types of ADEs, including ADEs originating from DAs.

CPOE systems can help in making fewer PEs, although not all studies quantify this improvement. Benkhaial et al [44] did not find a significant difference in the risk of being prescribed a drug potentially inducing an allergy using electronic recording of the allergy via ICD‑10 codes compared with paper records. Oliven et al [89] compared the number of PEs between a department using handwritten drug orders with that of a department using CPOE systems and found a reduced number of PEs in the department using CPOE. No significant difference was found between the two departments for DAs. On the contrary, Evans et al [54] demonstrated a reduction from 2.46% (28/1136) patients to 0.07% (4/545) patients with adverse events caused by anti-infective agents due to the introduction of a computerized anti-infective management program. Likewise Mahoney et al [56] demonstrated a reduction in PE rates related to DAs from 833 in the preimplementation phase to 109 in the postimplementation phase (OR 0.14, 95% CI 0.11-0.17).

When looking at the outcomes for ADEs, Bates et al [83] demonstrated a reduction of 55%, from 10.7 events per 1000 patient-days to 4.86 events per 1000 patient-days for nonintercepted serious medication errors, defined as those either resulting in or with the potential to result in ADEs. In a follow-up study [84], this rate decreased to 1.1 events per 1000 patient-days after additional refinements of the system. This objective was reached by including a dose selection menu, simple DA and DDI checking, and the requirement that clinicians indicate the route and frequency of drug administration. Hsieh et al [37] found that ADEs resulting from overridden DA alerts do not occur frequently (19/320, 5.9%). In this study, none of the ADEs were considered preventable because the overrides were deemed clinically justifiable. There is limited evidence that systems, mostly electronic systems combining CPOE with CDS, for preventing represcription after the occurrence of an ADE (including DAs) are effective [90].

The implementation of CDSS can also influence economic outcomes, eg, by decreasing costs related to medication errors [86,88]. However, information on cost-effectiveness, specifically of CDS for DAs, was not found.

## Discussion

### Principal Findings

To the best of our knowledge, this is the first systematic review focusing on CDSS in the field of DA. We included 69 articles in our review. The main findings are the problem of incomplete and inaccurate recording of patients’ DA information, the absence of an appropriate standard terminology that guides the rule bases within a CDSS, problems with rule bases, and the low specificity of DA alerts resulting in alert fatigue.

The first key finding was the incomplete or inaccurate documenting of patients’ DA information in medical records. Accurate and comprehensive recording of DA information in EHRs is essential for the proper functioning of CDSS for DA screening. A recurrent problem described in the literature is the absence of documented information on patients’ allergies, which can be interpreted in two ways: (1) the patient has no known allergies or (2) the patient has an allergy to a certain substance that has not yet been documented in the patient record [92]. Therefore, the absence of any known DA should also be documented in EHR. Besides underreporting of DAs, outdated or inaccurate DA information in EHRs also poses an important problem.

The second key finding was the absence of a generally accepted standard terminology for the structured documentation of allergy information. The use of Anatomical Therapeutic Chemical, ICD, NDF-RT, RxNorm, and SNOMED CT was described in the literature, but limited information was provided about the exact manner of implementation or integration of these coding systems. An evaluation of terminologies by Goss et al [48] showed that currently, a combination of RxNorm and SNOMED CT satisfies most criteria for encoding allergies. The use of free text for documenting DA information in EHRs should be discouraged because of the difficulties for CDS. The use of a standard terminology is required for coded exchange of DA information between institutions on a national and international level and for creating exchangeable decision rules based on standard terminologies. Governments have an important role in providing standardized terminologies in the official national languages. Policies and regulations may be required to support the effective use of coding standards in clinical practice.

The third key finding was that all reported CDSS for DA screening were knowledge-based systems requiring timely review of the rule bases to keep CDSS up to date. Ideally, end users and program developers should work together to regularly review the alerts logs and decision rules to reduce the risk of alert fatigue [67]. This is a continuous process and not a “one and done” step. Both in-house curated knowledge bases and vendor-based rule bases were reported in the literature, and both have their advantages and disadvantages. In an in-house curated knowledge base, flexibility is guaranteed, leading to potentially higher alert specificity, but it requires substantial effort to develop and maintain the rules base. A vendor-based rule base is easily purchased, but it has less flexibility when it comes to changing decision rules. The end user is dependent on the vendor for updates, but the maintenance burden lies with the vendor. A third possibility is the implementation of a hybrid system combining a commercial rule base with internally defined content refinements or decision rules.

The last key finding is the consistently low specificity of DA alerts. This remains an important problem as it causes high override rates, resulting in alert fatigue. Researchers have tried to tackle the problem of alert fatigue by providing on-demand decision support or customizable computer-triggered decision support. Another option is to turn off certain alerts, for example, by looking at the personal preferences of the healthcare provider who can decide to no longer receive a particular type of warning [93]. It remains difficult to find a good balance between reducing alert overload and keeping patient safety at a high level. A fixed rule base may therefore not always be appropriate; rather, an adaptive CDSS supported by a predictive risk model may be more useful [70]. Taking contextual factors into consideration as part of the CDS rules may help in increasing the specificity of DA alerts and lowering the rate of alert overrides. This strategy has been successfully applied for increasing the specificity of DDI alerts. Duke et al [94,95] and Cornu et al [96] have developed context-aware DDI alerts based on relevant patient-specific information, resulting in improved alert acceptance.

### Recommendations for Policy, Practice, and Future Research

Future policies should focus on the implementation of standard terminologies to allow standardized coded exchange of DA information on a national and international level and to create exchangeable decision rules.

Information on the effect of CDSS for DAs on patient outcomes was very limited. Thus, future research should focus on evaluating patient outcomes. Hsieh et al [37] demonstrated that after overriding DA alerts, none of the resulting ADEs were preventable. However, in their study, only overridden DA alerts were evaluated. It would be interesting to know the number of ADEs that was effectively prevented by CDSS.

It is assumed that implementing DA checking in a CPOE system also has a beneficial financial impact. We did not find any studies specifically related to the economic outcomes of CDS for DAs, but general conclusions about the economic benefits of implementing CPOE systems for the hospitals were documented. At the start, the implementation of a CPOE system requires a large investment, but soon the costs are outweighed by the benefits and result in savings [97]. However, the cost-effectiveness of CDSS for DA should be further investigated.

Current systems often warn about all possible cross-reactions, although the substance-specific risk should be estimated and the severity of the alert may change as a function of the possibility of cross-reaction (eg, likely, possible, or unlikely). Future research should explore strategies for optimizing cross-reactivity rules and enhancing alert specificity.

### Study Limitations

This study has several limitations. First, because of the heterogeneity across the study objectives, study designs, study populations, and reported results, a meta-analysis could not be performed. Second, different study designs require a different methodological framework for assessing bias. The heterogeneity in quantitative and qualitative study designs, reviews, and reports did not allow for a comprehensive and consistent evaluation of bias. This may limit the generalization of the results, but it allowed us to take a broader view of all relevant research in the field of CDS for DAs. Third, we excluded non-English papers, which may constitute selection bias. Additionally, 9 papers were added based on expert opinion because they included information relevant to this review. These extra articles were not retrieved with the query because they included keywords other than those included in the search query. Adjusting the query was not feasible because the keywords were often too general (eg, medication safety), which would result in a high number of irrelevant articles. Finally, publication bias cannot be excluded. We observed a high number of studies published in the US setting, which may lower the international relevance of the results. However, we believe that the findings of our review are relevant in an international context.

### Conclusions

This review shows that CPOE systems with CDS for DA screening are perceived as useful in clinical practice. Nevertheless, there are some important problems associated with their use. First, it is not yet clear how and where to document DA information in patients’ EHRs. Second, there is a lack of proper coding terminology for documenting allergies. A major problem with current systems is that alerts are not specific enough, resulting in high override rates and consecutive alert fatigue. Future research should focus on strategies to improve alert specificity and evaluating patient and economic outcomes.

## Appendix

Multimedia Appendix 1 Search strategy.

Multimedia Appendix 2 Table with author names, year of publication, study design, aim of the study, and key findings of articles included in the review.

Multimedia Appendix 3 Table with the number of subjects included in the review.

## Abbreviations

ADE: adverse drug event
ADR: adverse drug reaction
CDS: clinical decision support
CDSS: clinical decision support system(s)
CINAHL: Cumulative Index to Nursing and Allied Health Literature
CPOE: Computerized Physician Order Entry
DA: drug allergy
DDI: drug-drug interaction
EHR: electronic health record
ICD: International Classification of Diseases
NDF-RT: National Drug File-Reference Terminology
NLP: natural language processing
PE: prescribing error
OR: odds ratio
PRISMA: Preferred Reporting Items for Systematic Reviews and Meta-Analyses
SNOMED CT: Systematized Nomenclature of Medicine, Clinical Terms
UNII: unique ingredient identifier

## References

[1] Slight SP, Beeler PE, Seger DL, Amato MG, Her QL, Swerdloff M, Dalleur O, Nanji KC, Cho I, Maniam N, Eguale T, Fiskio JM, Dykes PC, Bates DW (2017). A cross-sectional observational study of high override rates of drug allergy alerts in inpatient and outpatient settings, and opportunities for improvement. BMJ Qual Saf.

[2] Adkinson Jr FN, Bochner BS, Burks AW, Busse WW, Holgate ST, Adkinson Jr N, Lemanske RF, O'Herir (2013). Middleton's Allergy Principles and Practice, Eight edition - volume 1.

[3] Gomes ER, Brockow K, Kuyucu S, Saretta F, Mori F, Blanca-Lopez N, Ott H, Atanaskovic-Markovic M, Kidon M, Caubet J, Terreehorst I, ENDA/EAACI Drug Allergy Interest Group (2016). Drug hypersensitivity in children: report from the pediatric task force of the EAACI Drug Allergy Interest Group. Allergy.

[4] Lee CE, Zembower TR, Fotis MA, Postelnick MJ, Greenberger PA, Peterson LR, Noskin GA (2000). The incidence of antimicrobial allergies in hospitalized patients: implications regarding prescribing patterns and emerging bacterial resistance. Arch Intern Med.

[5] Thong BY, Leong K, Tang C, Chng H (2003). Drug allergy in a general hospital: Results of a novel prospective inpatient reporting system. Ann Allergy Asthma Immunol.

[6] Park CS, Kim T, Kim SL, Kim JY, Yang KA, Bae Y, Cho YS, Moon H (2008). The use of an electronic medical record system for mandatory reporting of drug hypersensitivity reactions has been shown to improve the management of patients in the university hospital in Korea. Pharmacoepidemiol Drug Saf.

[7] Leape LL (1995). Preventing adverse drug events. Am J Health Syst Pharm.

[8] Bates DW, Boyle DL, Vander Vliet MB, Schneider J, Leape L (1995). Relationship between medication errors and adverse drug events. J Gen Intern Med.

[9] Bates DW, Cullen DJ, Laird N, Petersen LA, Small SD, Servi D, Laffel G, Sweitzer BJ, Shea BF, Hallisey R (1995). Incidence of adverse drug events and potential adverse drug events. Implications for prevention. ADE Prevention Study Group. JAMA.

[10] Lesar TS, Briceland L, Stein DS (1997). Factors related to errors in medication prescribing. JAMA.

[11] Bates DW, Spell N, Cullen DJ, Burdick E, Laird N, Petersen LA, Small SD, Sweitzer BJ, Leape LL (1997). The costs of adverse drug events in hospitalized patients. Adverse Drug Events Prevention Study Group. JAMA.

[12] Classen DC, Pestotnik SL, Evans RS, Lloyd JF, Burke JP (1997). Adverse drug events in hospitalized patients. Excess length of stay, extra costs, and attributable mortality. JAMA.

[13] Macy E, Contreras R (2014). Health care use and serious infection prevalence associated with penicillin “allergy” in hospitalized patients: A cohort study. J Allergy Clin Immunol.

[14] Kuperman GJ, Bobb A, Payne TH, Avery AJ, Gandhi TK, Burns G, Classen DC, Bates DW (2007). Medication-related clinical decision support in computerized provider order entry systems: a review. J Am Med Inform Assoc.

[15] Cufar A, Droljc A, Orel A (2012). Electronic medication ordering with integrated drug database and clinical decision support system. Stud Health Technol Inform.

[16] Kuperman GJ, Teich JM, Gandhi TK, Bates DW (2001). Patient safety and computerized medication ordering at Brigham and Women's Hospital. Jt Comm J Qual Improv.

[17] Topaz M, Seger DL, Lai K, Wickner PG, Goss F, Dhopeshwarkar N, Chang F, Bates DW, Zhou L (2015). High Override Rate for Opioid Drug-allergy Interaction Alerts: Current Trends and Recommendations for Future. Stud Health Technol Inform.

[18] Coleman JJ, van der Sijs H, Haefeli WE, Slight SP, McDowell SE, Seidling HM, Eiermann B, Aarts J, Ammenwerth E, Slee A, Ferner RE, Ferner RE, Slee A (2013). On the alert: future priorities for alerts in clinical decision support for computerized physician order entry identified from a European workshop. BMC Med Inform Decis Mak.

[19] Stultz JS, Nahata MC (2012). Computerized clinical decision support for medication prescribing and utilization in pediatrics. J Am Med Inform Assoc.

[20] Johnston ME, Langton KB, Haynes RB, Mathieu A (1994). Effects of computer-based clinical decision support systems on clinician performance and patient outcome. A critical appraisal of research. Ann Intern Med.

[21] Hunt DL, Haynes RB, Hanna SE, Smith K (1998). Effects of computer-based clinical decision support systems on physician performance and patient outcomes: a systematic review. JAMA.

[22] Kaushal R, Shojania KG, Bates DW (2003). Effects of computerized physician order entry and clinical decision support systems on medication safety: a systematic review. Arch Intern Med.

[23] Garg AX, Adhikari NKJ, McDonald H, Rosas-Arellano MP, Devereaux PJ, Beyene J, Sam J, Haynes RB (2005). Effects of computerized clinical decision support systems on practitioner performance and patient outcomes: a systematic review. JAMA.

[24] Chaudhry B, Wang J, Wu S, Maglione M, Mojica W, Roth E, Morton SC, Shekelle PG (2006). Systematic review: impact of health information technology on quality, efficiency, and costs of medical care. Ann Intern Med.

[25] Eslami S, de Keizer NF, Abu-Hanna A (2008). The impact of computerized physician medication order entry in hospitalized patients--a systematic review. Int J Med Inform.

[26] Schedlbauer A, Prasad V, Mulvaney C, Phansalkar S, Stanton W, Bates DW, Avery AJ (2009). What evidence supports the use of computerized alerts and prompts to improve clinicians' prescribing behavior?. J Am Med Inform Assoc.

[27] Shojania KG, Jennings A, Mayhew A, Ramsay CR, Eccles MP, Grimshaw J (2009). The effects of on-screen, point of care computer reminders on processes and outcomes of care. Cochrane Database Syst Rev.

[28] Brenner SK, Kaushal R, Grinspan Z, Joyce C, Kim I, Allard RJ, Delgado D, Abramson EL (2016). Effects of health information technology on patient outcomes: a systematic review. J Am Med Inform Assoc.

[29] Moher D, Liberati A, Tetzlaff J, Altman DG (2009). Preferred reporting items for systematic reviews and meta-analyses: the PRISMA statement. BMJ.

[30] Fernando B, Morrison Z, Kalra D, Cresswell K, Sheikh A (2014). Approaches to recording drug allergies in electronic health records: qualitative study. PLoS One.

[31] Mills DH (1964). Allergic reactions to drugs. A survey on hospital practices of soliciting medical information from newly admitted patients. Calif Med.

[32] Burrell C, Tsourounis C, Quan D, Jue V, Tam E, Guglielmo BJ, Bcps (2013). Impact of a pharmacist-driven protocol to improve drug allergy documentation at a university hospital. Hosp Pharm.

[33] Gay KJ, Hill C, Bell T (2009). Accuracy of drug-allergy recording in a district general hospital. Int J Pharm Pract.

[34] Mawby J (2006). Accurate documenting of a patient's drug allergy status will promote informed therapy decision-making. Pharmacy in Practice.

[35] Cresswell KM, Sheikh A (2008). Information technology-based approaches to reducing repeat drug exposure in patients with known drug allergies. J Allergy Clin Immunol.

[36] Ferner RE, Coleman JJ (2010). An algorithm for integrating contraindications into electronic prescribing decision support. Drug Saf.

[37] Hsieh TC, Kuperman GJ, Jaggi T, Hojnowski-Diaz P, Fiskio J, Williams DH, Bates DW, Gandhi TK (2004). Characteristics and consequences of drug allergy alert overrides in a computerized physician order entry system. J Am Med Inform Assoc.

[38] Lopez-Gonzalez E, Herdeiro MT, Figueiras A (2009). Determinants of under-reporting of adverse drug reactions: a systematic review. Drug Saf.

[39] Porter SC, Manzi SF, Volpe D, Stack AM (2006). Getting the data right: information accuracy in pediatric emergency medicine. Qual Saf Health Care.

[40] Rimawi RH, Shah KB, Cook PP (2013). Risk of redocumenting penicillin allergy in a cohort of patients with negative penicillin skin tests. J Hosp Med.

[41] Ross S, Ryan C, Duncan EM, Francis JJ, Johnston M, Ker JS, Lee AJ, MacLeod MJ, Maxwell S, McKay G, McLay J, Webb DJ, Bond C (2013). Perceived causes of prescribing errors by junior doctors in hospital inpatients: a study from the PROTECT programme. BMJ Qual Saf.

[42] Schiff GD, Rucker TD (1998). Computerized prescribing: building the electronic infrastructure for better medication usage. JAMA.

[43] Abookire SA, Teich JM, Sandige H, Paterno MD, Martin MT, Kuperman GJ, Bates DW (2000). Improving allergy alerting in a computerized physician order entry system. Proc AMIA Symp.

[44] Benkhaial A, Kaltschmidt J, Weisshaar E, Diepgen TL, Haefeli WE (2009). Prescribing errors in patients with documented drug allergies: comparison of ICD-10 coding and written patient notes. Pharm World Sci.

[45] Bernstein K (2014). Reporting of drug allergies for use in a national decision support system. Stud Health Technol Inform.

[46] Chaffee BW, Zimmerman CR (2010). Developing and implementing clinical decision support for use in a computerized prescriber-order-entry system. Am J Health Syst Pharm.

[47] Demner-Fushman D, Chapman WW, McDonald CJ (2009). What can natural language processing do for clinical decision support?. J Biomed Inform.

[48] Goss FR, Zhou L, Plasek JM, Broverman C, Robinson G, Middleton B, Rocha RA (2013). Evaluating standard terminologies for encoding allergy information. J Am Med Inform Assoc.

[49] Greibe K (2013). Development of a SNOMED CT based national medication decision support system. Stud Health Technol Inform.

[50] Ogallo W, Kanter AS (2015). Towards a Clinical Decision Support System for Drug Allergy Management: Are Existing Drug Reference Terminologies Sufficient for Identifying Substitutes and Cross-Reactants?. Stud Health Technol Inform.

[51] Paul L, Robinson KM (2012). Capture and documentation of coded data on adverse drug reactions: an overview. HIM J.

[52] Slight SP, Berner ES, Galanter W, Huff S, Lambert BL, Lannon C, Lehmann CU, McCourt BJ, McNamara M, Menachemi N, Payne TH, Spooner SA, Schiff GD, Wang TY, Akincigil A, Crystal S, Fortmann SP, Bates DW (2015). Meaningful Use of Electronic Health Records: Experiences From the Field and Future Opportunities. JMIR Med Inform.

[53] Baysari MT, Westbrook JI, Egan B, Day RO (2013). Identification of strategies to reduce computerized alerts in an electronic prescribing system using a Delphi approach. Stud Health Technol Inform.

[54] Evans RS, Pestotnik SL, Classen DC, Clemmer TP, Weaver LK, Orme JF, Lloyd JF, Burke JP (1998). A computer-assisted management program for antibiotics and other antiinfective agents. N Engl J Med.

[55] Kesselheim AS, Cresswell K, Phansalkar S, Bates DW, Sheikh A (2011). Clinical decision support systems could be modified to reduce 'alert fatigue' while still minimizing the risk of litigation. Health Aff (Millwood).

[56] Mahoney CD, Berard-Collins CM, Coleman R, Amaral JF, Cotter CM (2007). Effects of an integrated clinical information system on medication safety in a multi-hospital setting. Am J Health Syst Pharm.

[57] Ariosto D (2014). Factors Contributing to CPOE Opiate Allergy Alert Overrides. AMIA Annu Symp Proc.

[58] Brodowy B, Nguyen D (2016). Optimization of clinical decision support through minimization of excessive drug allergy alerts. Am J Health Syst Pharm.

[59] Bryant AD, Fletcher GS, Payne TH (2014). Drug interaction alert override rates in the Meaningful Use era: no evidence of progress. Appl Clin Inform.

[60] Carspecken CW, Sharek PJ, Longhurst C, Pageler NM (2013). A clinical case of electronic health record drug alert fatigue: consequences for patient outcome. Pediatrics.

[61] Dekarske BM, Zimmerman CR, Chang R, Grant PJ, Chaffee BW (2015). Increased appropriateness of customized alert acknowledgement reasons for overridden medication alerts in a computerized provider order entry system. Int J Med Inform.

[62] Falade O, Knight A, Maygers J, Sevransky JE (2012). Provider acceptance of medication warnings in a computerized provider order entry system. Am J Respir Crit Care Med.

[63] Genco EK, Forster JE, Flaten H, Goss F, Heard KJ, Hoppe J, Monte AA (2016). Clinically Inconsequential Alerts: The Characteristics of Opioid Drug Alerts and Their Utility in Preventing Adverse Drug Events in the Emergency Department. Ann Emerg Med.

[64] González-Gregori R, Dolores HFDRM, López-Salgueiro R, Díaz-Palacios M, García AN (2012). Allergy alerts in electronic health records for hospitalized patients. Ann Allergy Asthma Immunol.

[65] Horsky J, Schiff GD, Johnston D, Mercincavage L, Bell D, Middleton B (2012). Interface design principles for usable decision support: a targeted review of best practices for clinical prescribing interventions. J Biomed Inform.

[66] Hunteman L, Ward L, Read D, Jolly M, Heckman M (2009). Analysis of allergy alerts within a computerized prescriber-order-entry system. Am J Health Syst Pharm.

[67] Jani YH, Barber N, Wong ICK (2011). Characteristics of clinical decision support alert overrides in an electronic prescribing system at a tertiary care paediatric hospital. Int J Pharm Pract.

[68] Knight AM, Falade O, Maygers J, Sevransky JE (2015). Factors associated with medication warning acceptance for hospitalized adults. J Hosp Med.

[69] Kuperman GJ, Gandhi TK, Bates DW (2003). Effective drug-allergy checking: methodological and operational issues. J Biomed Inform.

[70] Lee EK, Wu T, Senior T, Jose J (2014). Medical alert management: a real-time adaptive decision support tool to reduce alert fatigue. AMIA Annu Symp Proc.

[71] Lin C, Payne TH, Nichol WP, Hoey PJ, Anderson CL, Gennari JH (2008). Evaluating clinical decision support systems: monitoring CPOE order check override rates in the Department of Veterans Affairs' Computerized Patient Record System. J Am Med Inform Assoc.

[72] Lopez R, Gonzalez R, Hernandez D, Hervas D, Campos A, Diaz M (2012). Allergy alerts in hospital electronic medical records. Special Issue: XXXI Congress of the European Academy of Allergy and Clinical Immunology Abstract Book, Geneva, Switzerland, 16‐20 June 2012.

[73] McCoy AB, Thomas EJ, Krousel-Wood M, Sittig DF (2014). Clinical decision support alert appropriateness: a review and proposal for improvement. Ochsner J.

[74] Nanji KC, Slight SP, Seger DL, Cho I, Fiskio JM, Redden LM, Volk LA, Bates DW (2014). Overrides of medication-related clinical decision support alerts in outpatients. J Am Med Inform Assoc.

[75] Russ AL, Zillich AJ, Melton BL, Russell SA, Chen S, Spina JR, Weiner M, Johnson EG, Daggy JK, McManus MS, Hawsey JM, Puleo AG, Doebbeling BN, Saleem JJ (2014). Applying human factors principles to alert design increases efficiency and reduces prescribing errors in a scenario-based simulation. J Am Med Inform Assoc.

[76] Shah NR, Seger AC, Seger DL, Fiskio JM, Kuperman GJ, Blumenfeld B, Recklet EG, Bates DW, Gandhi TK (2006). Improving acceptance of computerized prescribing alerts in ambulatory care. J Am Med Inform Assoc.

[77] Swiderski SM, Pedersen CA, Schneider PJ, Miller AS (2017). A Study of the Frequency and Rationale for OverridingAllergy Warnings in a Computerized Prescriber Order Entry System. J Patient Saf.

[78] Tamblyn R, Huang A, Taylor L, Kawasumi Y, Bartlett G, Grad R, Jacques A, Dawes M, Abrahamowicz M, Perreault R, Winslade N, Poissant L, Pinsonneault A (2008). A randomized trial of the effectiveness of on-demand versus computer-triggered drug decision support in primary care. J Am Med Inform Assoc.

[79] Taylor LK, Tamblyn R (2004). Reasons for physician non-adherence to electronic drug alerts. Stud Health Technol Inform.

[80] Topaz M, Seger DL, Slight SP, Goss F, Lai K, Wickner PG, Blumenthal K, Dhopeshwarkar N, Chang F, Bates DW, Zhou L (2016). Rising drug allergy alert overrides in electronic health records: an observational retrospective study of a decade of experience. J Am Med Inform Assoc.

[81] Weingart SN, Simchowitz B, Shiman L, Brouillard D, Cyrulik A, Davis RB, Isaac T, Massagli M, Morway L, Sands DZ, Spencer J, Weissman JS (2009). Clinicians' assessments of electronic medication safety alerts in ambulatory care. Arch Intern Med.

[82] Weingart SN, Massagli M, Cyrulik A, Isaac T, Morway L, Sands DZ, Weissman JS (2009). Assessing the value of electronic prescribing in ambulatory care: a focus group study. Int J Med Inform.

[83] Bates DW, Leape LL, Cullen DJ, Laird N, Petersen LA, Teich JM, Burdick E, Hickey M, Kleefield S, Shea B, Vander VM, Seger DL (1998). Effect of computerized physician order entry and a team intervention on prevention of serious medication errors. JAMA.

[84] Bates DW, Teich JM, Lee J, Seger D, Kuperman GJ, Ma'Luf N, Boyle D, Leape L (1999). The impact of computerized physician order entry on medication error prevention. J Am Med Inform Assoc.

[85] Beccaro MAD, Villanueva R, Knudson KM, Harvey EM, Langle JM, Paul W (2010). Decision Support Alerts for Medication Ordering in a Computerized Provider Order Entry (CPOE) System: A systematic approach to decrease alerts. Appl Clin Inform.

[86] Fung KW, Vogel LH (2003). Will decision support in medications order entry save money? A return on investment analysis of the case of the Hong Kong hospital authority. AMIA Annu Symp Proc.

[87] Harolds JA, Harolds LB (2016). Quality and Safety in Health Care, Part IX: Computerized Provider Order Entry. Clin Nucl Med.

[88] Leu W, Chen H, Chien H, Liu H, Chiueh C, Lin YM (2013). The financial impact of computer systems-based approaches to reducing repeat drug exposure in patients with known drug allergies. Int J Clin Pharmacol Ther.

[89] Oliven A, Michalake I, Zalman D, Dorman E, Yeshurun D, Odeh M (2005). Prevention of prescription errors by computerized, on-line surveillance of drug order entry. Int J Med Inform.

[90] van der Linden CM, Jansen PAF, Grouls RJE, van Marum RJ, Verberne MAJW, Aussems LMA, Egberts TCG, Korsten EHM (2013). Systems that prevent unwanted represcription of drugs withdrawn because of adverse drug events: a systematic review. Ther Adv Drug Saf.

[91] Varkey P, Aponte P, Swanton C, Fischer D, Johnson SF, Brennan MD (2007). The effect of computerized physician-order entry on outpatient prescription errors. Manag Care Interface.

[92] Hayes BD, Donovan JL, Smith BS, Hartman CA (2007). Pharmacist-conducted medication reconciliation in an emergency department. Am J Health Syst Pharm.

[93] van der Sijs H, Aarts J, Vulto A, Berg M (2006). Overriding of drug safety alerts in computerized physician order entry. J Am Med Inform Assoc.

[94] Duke JD, Bolchini D (2011). A successful model and visual design for creating context-aware drug-drug interaction alerts. AMIA Annu Symp Proc.

[95] Duke JD, Li X, Dexter P (2013). Adherence to drug-drug interaction alerts in high-risk patients: a trial of context-enhanced alerting. J Am Med Inform Assoc.

[96] Cornu P, Steurbaut S, Gentens K, Van de Velde R, Dupont AG (2015). Pilot evaluation of an optimized context-specific drug-drug interaction alerting system: A controlled pre-post study. Int J Med Inform.

[97] Kaushal R, Jha AK, Franz C, Glaser J, Shetty KD, Jaggi T, Middleton B, Kuperman GJ, Khorasani R, Tanasijevic M, Bates DW, BrighamWomen's HCWG (2006). Return on investment for a computerized physician order entry system. J Am Med Inform Assoc.

