# Supplementary material for: Clinical Decision Support Systems for Drug Allergy Checking: Systematic Review
Source: J Med Internet Res. 2018 Sep 7;20(9):e258. doi: 10.2196/jmir.8206 (PMC6231757; doi:10.2196/jmir.8206)
Supplement: Multimedia Appendix 1 [file jmir_v20i9e258_app1.pdf]

GENERAL FLOW

Step 1
Search CPOE:
- “computerized physician order entry”
- “computerized provider order entry”
- “computerized prescriber order entry”
- “computerized order entry”
- “computerised physician order entry”
- “computerised provider order entry”
- “computerised prescriber order entry”
- “computerised order entry”
- “electronic prescribing”
- “electronic prescription”
- “electronic physician order entry”

Step 2
Search CDSS:
- “clinical decision support”
- “clinical decision making”
- “decision support”

Step 3
Search *Step 1* OR *Step2*

CPOE OR CDSS

Step 4
Search Alert*:
- “alert”
- “alerting”

Step 5
Search *Step 3* OR *Step 4*

(CPOE OR CDSS) OR Alert*

Step 6
Search Allerg*:
- “allergic”
- “allergy”

Step 7
Search (CPOE OR CDSS OR Alert*) AND *Step 6*

(CPOE OR CDSS OR Alert*) AND Allerg*

FLOW PER DIGITAL LIBRARY

CINAHL

Step 1
“computerized physician order entry” OR “computerized provider order entry” OR “computerized prescriber order entry” OR “computerized order entry” OR “computerised physician order entry” OR “computerised provider order entry” OR “computerised prescriber order entry” OR “computerised order entry” OR “electronic prescribing” OR “electronic prescription” OR “electronic physician order entry”
Step 2
“clinical decision support” OR “clinical decision making” OR “decision support”
Step 3
Step 1 OR *Step 2*
Step 4
“alert*”
Step 5
Step 3 OR *Step 4*
Step 6
“allerg*”
Step 7
Step 5 AND Step 6

COCHRANE

Step 1
“computerized physician order entry” OR “computerized provider order entry” OR “computerized prescriber order entry” OR “computerized order entry” OR “computerised physician order entry” OR “computerised provider order entry” OR “computerised prescriber order entry” OR “computerised order entry” OR “electronic prescribing” OR “electronic prescription” OR “electronic physician order entry”
Step 2
“clinical decision support” OR “clinical decision making” OR “decision support”
Step 3
Step 1 OR *Step 2*
Step 4
“alert*”
Step 5
Step 3 OR *Step 4*
Step 6
“Allergic” OR “Allergy”
Step 7
Step 5 AND Step 6

EMBASE

Step 1

'computerized physician order entry':ab,ti OR ‘computerized provider order entry’:ab,ti OR ‘computerized prescriber order entry’:ab,ti OR ‘computerized order entry’:ab,ti OR ‘computerised physician order entry’:ab,ti OR ‘computerised provider order entry’:ab,ti OR ‘computerised prescriber order entry’:ab,ti OR ‘computerised order entry’:ab,ti OR ‘electronic prescribing’:ab,ti OR ‘electronic prescription’:ab,ti OR ‘electronic physician order entry’:ab,ti

Step 2

‘clinical decision support’:ab,ti OR ‘clinical decision making’:ab,ti OR ‘decision support’:ab,ti

Step 3

Step 1 OR *Step 2*

Step 4

“alert*”

Step 5

Step 3 OR *Step 4*

Step 6

‘allerg*’:ab,ti

Step 7

Step 5 AND *Step 6*

‘allerg*’:ab,Ti

OVID

Step 1

“computerized physician order entry” OR “computerized provider order entry” OR “computerized prescriber order entry” OR “computerized order entry” OR “computerised physician order entry” OR “computerised provider order entry” OR “computerised prescriber order entry” OR “computerised order entry” OR “electronic prescribing” OR “electronic prescription” OR “electronic physician order entry”

Step 2

“clinical decision support” OR “clinical decision making” OR “decision support”

Step 3

Step 1 OR *Step 2*

Step 4

“alert*”

Step 5

Step 3 OR *Step 4*

Step 6

“allerg*”

Step 7

Step 5 AND Step 6

PUBMED

Step 1
"computerized physician order entry"[Title/Abstract] OR "computerized provider order entry"[Title/Abstract] OR "computerized prescriber order entry"[Title/Abstract] OR "computerized order entry"[Title/Abstract] OR "computerised physician order entry"[Title/Abstract] OR "computerised provider order entry"[Title/Abstract] OR "computerised prescriber order entry"[Title/Abstract] OR "computerised order entry"[Title/Abstract] OR "electronic prescribing"[Title/Abstract] OR "electronic prescription"[Title/Abstract] OR "electronic physician order entry"[Title/Abstract]
Step 2
"clinical decision support"[Title/Abstract] OR "clinical decision making"[Title/Abstract] OR "decision support"[Title/Abstract]
Step 3
Step 1 OR *Step 2*
Step 4
"alert"[Title/Abstract] OR "alerting"[Title/Abstract]
Step 5
Step 3 OR *Step 4*
Step 6
“allergy”[Title/Abstract] OR “allergic”[Title/Abstract]
Step 7
Step 5 AND Step 6
