# Supplementary material for: Clinical Decision Support Systems for Drug Allergy Checking: Systematic Review
Source: J Med Internet Res. 2018 Sep 7;20(9):e258. doi: 10.2196/jmir.8206 (PMC6231757; doi:10.2196/jmir.8206)
Supplement: Multimedia Appendix 2 [file jmir_v20i9e258_app2.pdf]

Multimedia Appendix 2. Table with author names, year of publication, study design, aim of the study, and key findings of articles included in the review.

Authors (year)	Study design	Aim	Key findings
DOCUMENTING THE PRESENCE/ABSENCE OF AN ALLERGY			
Burrell et al. (2013)	Before and after interventional study	Evaluate the impact of a pharmacist-driven protocol on the quality of drug allergy or drug intolerance documentation	The frequency of complete[a] DA/intolerance documentation raised from 52%-62% to 60%-76% by implementing a pharmacist-driven protocol. Pediatric services demonstrated the most substantial improvement, increasing from 53%-79% to 67%-93%. Blank reaction fields decreased by 10% in both groups. In general, a pharmacy-driven initiative improved the completeness of DA/intolerance documentation.
Cresswell et al. (2008)	Narrative review	Provide an overview of information technology-based interventions that can be used to reduce the risk of recurrent exposure of drug allergies	Information technology-based interventions that can be used to reduce the risk of recurrent exposure of drug allergies are:<br>- computer systems that incorporate hazard messages that alert healthcare professionals to patients' allergies.<br>- bar-coded wristbands to provide more comprehensive patient-specific information on the class of drug to which the patient is allergic.<br>- radiofrequency identification technology, which allows the storage of information (e.g., drug allergies).<br>- biometric technologies, such as fingerprint, face, or iris scanning can facilitate patient identification (and therefore identification of potential drug allergies).<br>- patient-generated and patient-managed electronic health records where patients are involved in the care process.
Fernando et al. (2014)	Practice experience	Investigate the variation in the way drug allergies are recorded in the electronic health record (EHR) of the patient	The DA information recorded and coded in practice varies depending on the purpose of recording and the nature of the tools available for recording. Although healthcare professionals widely appreciate

			the importance of recording this information there is at present no agreement amongst clinicians on what needs to be recorded in EHRs and how.
Ferner et al. (2010)	Exploratory, draft scheme for algorithm	Construct an algorithm that would classify contraindications according to the data available to a computerized system, and allow them to be displayed in context as far as possible	Contraindications can be used to classify alerts so that the warnings are only shown where relevant information was available. Most contraindications refer to current or past co-morbid conditions. If these are to be used by decision support systems they will have to be described in standard codable terms (e.g. SNOMED CT or ICD). Only systems with access to clinical data will be optimal, although even simple demographic data can be used to reduce the burden of unnecessary alerts.
Gay et al. (2009)	Observational retrospective study	Audit the accuracy of drug-allergy documentation based on the information provided by the patient	The majority of drug cards in the hospital had the allergy section filled in to some degree. The documentation of date and a subsequent signature was done poorly, along with the filling-in of the alert sheet stating the potential drug allergies and hypersensitivities.
Hsieh et al. (2004)	Observational retrospective study	Determine characteristics of drug allergy alert overrides, assess how often they lead to preventable adverse drug events (ADEs), and suggest methods for improving the allergy alerting system	80% of the DA alerts were overridden. The most frequent reason for override was "Aware/will monitor" (55%).<br>6% of the overrides resulted in an ADE, but none of them were life-threatening or fatal. Recommendation to only send a trigger for alert when there is an exact match between the ordered drug and allergy list.
Kuperman et al. (2007)	Narrative review	Assess some of the challenges associated with both basic and advanced decision support and discuss how those challenges might be addressed	Alerts should be clear and concise so that a care provider understands the rationale of the interruption. CDS knowledge ideally combines commercial rule bases, rules from organizations and rules about best practice. Healthcare organizations should prioritize in the long list of CDS features.

Lopez-Gonzalez et al. (2009)	Systematic review	Assess the influence of personal and professional characteristics on adverse drug reaction (ADR) reporting and to identify knowledge and attitudes associated with ADR reporting	Medical specialty was the professional characteristic most closely associated with under-reporting of adverse drug reactions in 76% of studies involving physicians. Other factors associated with under-reporting were:<br>- ignorance in 95% of the studies<br>- diffidence in 72% of the studies<br>- lethargy in 77% of the studies<br>- indifference and insecurity in 67% of the studies<br>- complacency in 47% of the studies<br>Moreover also knowledge and attitudes of health professionals appear to be strongly related with reporting in a high proportion of studies.
Mawby (2006)	Observational retrospective study	Audit the drug allergy records in two oncology wards at Southampton University Hospitals Trust to evaluate the accuracy of drug allergy documentation	In one ward the DA documentation on drug charts and in medical notes was 100% consistent. However, in the other ward only 82.4% of drug charts had an allergy entry on the drug chart, and of these only 68.8% (57.2% of the total) corresponded to information in the medical notes. This raises cause for concern because inaccuracies could lead to potentially serious and unnecessary allergic drug reactions.
Mills (1964)	Cross-sectional (retrospective)	Obtain information about the information that is captured in hospitals about allergies and drugs of the patients	Designated employees of 69% and 38.5% of the reporting hospitals require respectively obtaining information on allergies of newly admitted patients and on drugs recently taken. Only recently drug interaction received increasing attention.<br>74.7% and 56.3% of hospitals actually register respectively information on allergies and drugs. It is recommended that the task of inquiring is limited to medical and nursing personnel. With the accumulated information derived from this survey a checklist was composed that can serve as guideline.

Porter et al. (2006)	Observational study (direct observation at triage, structured verbal interview with parents to ascertain a full allergy history related to medications, and retrospective chart review)	- Identify the extent to which information provided by parents in the pediatric ED can drive the assessment and categorization of data on allergies to medications<br>- Identify errors related to the capture and documentation of allergy data at specific process level steps during ED care	Of the 256 observed parent-child dyads:<br>- 211 (82.4%) completed the structured verbal interview that served as the basis for the comparative standard (CS)<br>- 59 medications were reported by parents as possible allergies, of which 56 (94.9%) were categorized as allergy or not based on the CS. 28 of 48 patient cases were true allergies by guideline based assessment.<br>- sensitivity of triage for detecting true medication allergy was 74.1%. Specificity of triage personnel for correctly determining that no allergy existed was 93.2%<br>- physician and nursing care had performance gaps related to medication allergy in 10–25% of cases.
Rimawi et al. (2013)	Observational retrospective study	Assess the prevalence of reinstating inaccurate allergy information and associated factors thereof	Of the 150 patients with negative penicillin test:<br>- 55 (37%) revisited a Vidant system hospital within a 1-year period of whom 21 were long-term care facilities residents<br>- 22 (36%) of the 55 patients had penicillin allergy redocumented without apparent reason<br><br>Factors associated with penicillin allergy redocumentation included:<br>- age >65 years<br>- long-term care facilities residence<br>- acutely altered mentation<br>- dementia
Ross et al. (2013)	Observational prospective study (data collection) combined with interview transcript	Investigate the perceived causes of prescribing errors among foundation (junior) doctors in Scotland	Prescribing errors were categorized into five categories of error-producing conditions:<br>- work environment: workload and time pressures<br>- team factors: included multiple individuals and teams involved with a patient, poor communication, poor medicines reconciliation and documentation and following incorrect

			instructions from other members of the team or the assumption that another member of the team would identify any errors made<br>- individual factor: lack of personal knowledge and experience<br>- task factor: poor availability of drug information at admission<br>- patient factor: complexity
Schiff et al. (1998)	Narrative review	Outline areas in which computer prescribing might transform care processes and patient outcomes	- Choosing drugs should take recommendations as a guideline<br>- Therapy should evolve to a dialogue between patient, provider and computer instead of considering the patient as a passive actor<br>- Screening for drug-drug, drug-allergy and drug-disease interactions requires reliable software and information<br>- Link pharmacy and laboratory information<br>- Prescribing and scheduling of medication go hand-in-hand<br>- Medication documentation among care team members should be better coordinated, education can help here<br>- Computers help in facilitating adverse effect documentation<br>- Capturing feedback, e.g. when drug treatment is discontinued, can help in the care process
CODING			
Abookire et al. (2000)	Alerts and alert fatigue, Coding Observational retrospective study	Evaluate trends of allergy alerting and user response to allergy alerts	An increasing trend of DA alerting was observed. Over time, decreasing allergy alert compliance was found. This raises questions concerning the alert quality. Reviewing new alert rules is therefore important.
Benkhaial et al. (2009)	Retrospective cohort study	Compare quality of handwritten allergy information with electronic coding of this information and assess value of International Classification of Diseases, version 10 (ICD-10) codes for drug-allergies as triggers for clinical decision support	There was no difference in number of prescribing errors due to drugs potentially inducing allergies when the allergy was only documented as an ICD-10 code or the information was available in the paper record ($P > 0.05$).<br>This analysis suggests that

			optimizing the code system and encouraging to document allergies may help in preventing prescribing errors and decreasing the over-alerting rate.
Bernstein (2014)	Survey combined with telephone interviews and retrospective analysis of free text allergy records	Assess the possibility for reusing existing data by letting clinicians structure the data	One of the key challenges in trying to standardize data across sectors and professional groups is that a common definition is lacking. Systemized Nomenclature of Medical Clinical Terms (SNOMED CT) can help because of the extensive character and possibility to subset definition creation.
Chaffee et al. (2010)	Practice experience	Describe the implementation strategy, experience, and future plans for CDS at the University of Michigan Health System	Passive CDS considers: the nomenclature used, links to information, relevant results, and order sets.<br>Active CDS considers both non-interruptive alerts (patient list alert flags and form-called medical logic modules) and interruptive alerts, including alerts for allergies, dose checks, drug-drug interactions, drug-food interactions, and drug-disease interactions.<br>Challenges ahead are how to govern the existing and future CDS activities and decisions, to monitor the effectiveness of CDS and think about possible enhancements to the system.
Demner-Fushman et al. (2009)	Narrative review	Review the current practices of Natural Language Processing (NLP) and look at the possible applications in the clinical field	The past 45 years of natural language processing (NLP), systems and methods for CDS have not been translated into widespread use and daily practice. A problem is that most of the systems are developed only for one specific type of user. The strong foundation and local successes combined with the renewed community-wide interest to medical language processing provide hope that mature NLP systems for CDS will become available to the wider community in the near future. NLP can contribute to decision support for all groups involved in

			the clinical process, but the development will probably focus on the areas for which there is higher demand.
Goss et al. (2013)	Comparative study	Analyze and compare various existing standard terminologies for representing allergy information	The terminologies SNOMED CT, National Drug File - Reference Terminology (NDF-RT), Medication Dictionary for Regulatory Activities (MedDRA), Unique Ingredient Identifier (UNII) and RxNorm were compared both quantitatively and qualitatively. The results suggest that while gaps still exist, a combination of SNOMED CT and RxNorm can satisfy most criteria for encoding common allergies and providing sufficient content coverage. A criteria in which SNOMED CT outperformed all others was the ability to represent no known allergies for a patient.
Greibe (2013)	Case description	Introduce central decision support to both general practitioners and hospitals with a centralized platform and five new services in Denmark	Introducing clinical decision support, based on a structured terminology as SNOMED CT, will bring several benefits:<br>- improved patient treatment.<br>- decreased hospitalization time<br>- decreased health expenditures.<br>- peace of mind for the physician not having to check all parameters manually, which results in more time for the patient.<br>- a central allergy register will ensure allergies detected in e.g. the hospital will cause an alert at the general practitioner as well, without any action required by the general practitioner.<br>- possibility to control the level of alerts physicians are comfortable with, which should prevent alert fatigue.
Ogallo et al. (2015)	Practice experience	Assess the feasibility of using RxNorm and National Drug File - Reference Terminology (NDF-RT) for allergy management decision support	In the absence of an established medication allergy classification system, using the Pharmacologic Class and Chemical Structure classifications in NDF-RT may still be effective for discriminating between safe and cross-reactive

			alternatives for potential allergens.<br>RxNorm was used for identifying the ingredients for a number of drugs.
Paul et al. (2012)	Narrative review	Presents an overview of the nature and extent of adverse drug reactions (ADRs) in Australia and other developed countries.	ADRs constitute a largely preventable problem that imposes substantial morbidity, mortality and financial costs on individuals, hospitals and the community. The availability and reliability of ADR statistics can be compromised by irregularities in data collection and patient-related documentation, and prescribing errors resulting in ADRs, especially DA-related, often results from poor medical record documentation, using the International Classification of Diseases as coding standard.
Slight et al. (2015)	Focus group study	Examine the impact of meaningful use (MU) policy related to the use of medications and jointly developed recommendations to help inform future HIT	Professionals' knowledge and experiences from implementing meaningful use (MU) requirements fell into 6 domains:<br>- accuracy of medication lists and medication reconciliation<br>- problem list accuracy and the shift in health information technology (HIT) priorities<br>- accuracy of allergy lists and allergy-related standards development<br>- support of safer and effective prescribing for children<br>- considerations for rural communities<br>- general issues with achieving MU<br>Standards are needed to better facilitate the exchange of data elements between health care settings. Several organizations felt that their preoccupation with fulfilling MU requirements stifled innovation. Greater emphasis should be placed on local HIT configurations that better address population health care needs.

RULE BASES			
Baysari et al. (2013)	Survey (Delphi technique)	To reach consensus among prescribers on the usefulness of computerized alerts and strategies for reducing low-value alerts	Prescribers have different views on alerts but agree that allergy and intolerance alerts should be retained. Duplication of alerts does not comprise patient safety. Involving users in customization of alerts proved to be a successful approach.
Chaffee et al. (2010)	Practice experience	Describe the implementation strategy, experience, and future plans for CDS at the University of Michigan Health System	Passive CDS considers: the nomenclature used, links to information, relevant results, and order sets.<br>Active CDS considers both non-interruptive alerts (patient list alert flags and form-called medical logic modules) and interruptive alerts, including alerts for allergies, dose checks, drug-drug interactions, drug-food interactions, and drug-disease interactions.<br>Challenges ahead are how to govern the existing and future CDS activities and decisions, to monitor the effectiveness of CDS and think about possible enhancements to the system.
Evans et al. (1998)	Before and after interventional study	Study the use of a computerized decision-support program for the use of antibiotics and other anti-infective agents	The use of the computer-assisted management program for antibiotics and other anti-infective agents led to significant reductions in orders for drugs to which the patients had reported allergies, excess drug dosages, and antibiotic-susceptibility mismatches ($P < 0.01$). There were also marked reductions in the mean number of days of excessive drug dosage ($P < 0.01$) and in adverse events caused by anti-infective agents ($P = 0.02$).
Kesselheim et al. (2011)	Narrative review	Review the principles of product liability to examine whether fears of liability arising from medication-related clinical decision support systems (CDSS) are well grounded, viewed from the standpoint of designers and users	Parsimonious or tailored warnings are overstated, they do not raise the liability risk of system manufacturers and physicians as long as systems are designed well and providers continue to use their best medical judgment.<br>The legal situation surrounding CDSS is unclear. Stronger

			government regulation could help to lower both liability risk and perceptions of risk.
Kuperman et al. (2007)	Narrative review	Assess some of the challenges associated with both basic and advanced decision support and discuss how those challenges might be addressed	Alerts should be clear and concise so that a care provider understands the rationale of the interruption. CDS knowledge ideally combines commercial rule bases, rules from organizations and rules about best practice. Healthcare organizations should prioritize in the long list of CDS features.
Mahoney et al. (2007)	Before and after interventional study	Implementation of vendor-based integrated clinical information technology was studied, and its effect on medication errors throughout the medication-use process in a health care system was evaluated.	A positive effect of implementation of an integrated clinical information system on medication errors throughout the medication-use process was demonstrated.<br>Most prescribing errors decreased significantly in the selected categories monitored, specifically DA detection (OR=0.14, [95% CI 0.11-0.17]), excessive dosing (OR=0.68, [95% CI 0.62-0.74]), and incomplete or unclear orders (OR=0.35, [95% CI 0.32-0.38]) (P < 0.001).
ALERTS AND ALERT FATIGUE			
Abookire et al. (2000)	Alerts and alert fatigue, Coding Observational retrospective study	Evaluate trends of allergy alerting and user response to allergy alerts	An increasing trend of DA alerting was observed. Over time, decreasing allergy alert compliance was found. This raises questions concerning the alert quality. Reviewing new alert rules is therefore important.
Ariosto (2014)	Observational retrospective study	Identify factors that contribute to high volumes of low value alerts that are consistently overridden	The effect of several factors (e.g. age, race, gender, ...) on the likelihood of provider override of the patients' first opiate alert was analyzed using generalized estimating equations. Only prescriber role had a significant effect on alert override behavior, where physicians were more likely to override an alert compared to advanced practice nurses.
Baysari et al. (2013)	Survey (Delphi technique)	To reach consensus among prescribers on the usefulness of computerized alerts and strategies for reducing low-value	Prescribers have different views on alerts but agree that allergy and intolerance alerts should be retained. Duplication of alerts

		alerts	does not comprise patient safety. Involving users in customization of alerts proved to be a successful approach.
Brodowy et al. (2016)	Before and after interventional study	Reduce alert fatigue by optimizing drug allergy alerts	DA alert override rate was compared before and after the implementation of computerized provider order entry applications (drug-drug-interaction-, drug-allergy-, drug-dose- and duplicate therapy-checking) with CDS to handwritten medication orders without CDS.<br>The override rate for DA alerts dropped from 94% to 90% in the post-evaluation period; in other words, the acceptance rate for allergy alerts increased from 6% to 10%.<br>As of December 2015, the override rate for DA alerts continued to improve and was 80% at the end of the study.
Bryant et al. (2014)	Observational retrospective study	Measure override rates of alerts and compare override rates of DDI alerts and DAI alerts	Override rates remain as high as before (i.e. 92.9% overall).<br>Override rates were high in all categories, but the drug-drug interaction alert override rate was significantly higher than that for drug-allergy alerts (95.1% vs. 90.9%, $P < 0.001$).
Carspecken et al. (2013)	Case report	Present a case report in pediatrics where clinical decision support throws too many inappropriate DA alerts and propose a solution for this problem by working together with the electronic health record vendor	Alert fatigue encompasses many sociotechnical aspects of clinical health systems (proper education on how to document, incorporating evidenced-based medicine practices in health information systems and unifying medication allergies in one single digital location).<br>Turning off improper alerts is challenging because of variation in physician drug-related knowledge and hospital drug-monitoring routines. Uncertainty about drug reaction severity, legal concerns, and regulatory mandates with public reporting implications further complicate implementation of smarter alerting systems.

Chaffee et al. (2010)	Practice experience	Describe the implementation strategy, experience, and future plans for CDS at the University of Michigan Health System	Passive CDS considers: the nomenclature used, links to information, relevant results, and order sets.<br>Active CDS considers both non-interruptive alerts (patient list alert flags and form-called medical logic modules) and interruptive alerts, including alerts for allergies, dose checks, drug-drug interactions, drug-food interactions, and drug-disease interactions.<br>Challenges ahead are how to govern the existing and future CDS activities and decisions, to monitor the effectiveness of CDS and think about possible enhancements to the system.
Coleman et al. (2013)	Workshop	Assess current knowledge on alerts in CDS and reach a consensus on a future research agenda on this topic	Knowledge gaps that should be resolved in the future:<br>- where to find the optimum sensitivity and specificity of a CDS system in practice?<br>- will personalization of alerts reduce alert fatigue?<br>- will appropriate timing of an alert reduce alert fatigue?<br>- what outcome measure(s) should be used in evaluating alerts?
Dekarske et al. (2015)	Prospective randomized crossover study	Examine the effect of customizing medication alert override options per type of alert on the appropriateness of override selection related to patient allergies, drug dosing, and DDIs when ordering medications in an electronic medical record	Customized configuration (where a subdivision was made between DA, dose range and drug-drug interaction as override reason) had a higher rate of appropriateness of alert override responses when compared to the non-customized configuration (into which all override reasons were grouped) regardless of how indeterminate responses were classified ($P < 0.001$). One exception was found when indeterminate responses were considered inappropriate for the cohort of providers that were first exposed to the non-customized list ($P = 0.103$).<br>Free-text response use was higher in the customized configuration overall ($P < 0.001$),

			and there was no difference in nonsensical response between configurations (*P* = 0.39).
Falade et al. (2012)	Observational retrospective study	Observe the type of warnings and investigate what factors influence the alert acceptance	Only 3% of generated warnings were accepted. The largest category of warnings accepted were drug interactions (48%); closely followed by duplicates (39%) and then allergies (12%). Factors associated with acceptance of warnings include the type of warning and the level of training of a physician. Allergy warnings were most likely to be accepted (P < 0.001) and attending physicians were most likely to accept medication warnings (P = 0.003).
Genco et al. (2016)	Observational retrospective study	Determine characteristics of opioid drug alerts in the emergency department (ED)	The total proportion of overridden alerts was 93.5%. Opioid drug alerts were more likely to be overridden than non-opioid alerts (RR = 1.35, [95% CI 1.21-1.50]). Opioid drug-allergy alerts were twice as likely to be overridden than non-opioid alerts (RR = 2.24, [95% CI 1.74-2.89]). 14/4,581 patient visits had ADE of which 8 were attributed to opioid. None of these were preventable by clinical decision support.
González-Gregori et al. (2012)	Observational retrospective study	Evaluate the use of allergy alert entries in the EHR and to establish the allergy profile of hospitalized patients	2,660 alerts for allergy or intolerance related to:<br>- drugs (1,980 / 74.4%)<br>- food (336 / 12.6%)<br>- materials (129 / 4.8%)<br>The frequency of allergy alerts varies depending on age and sex. The study has demonstrated that the allergy alert is an underused resource. The most likely reason of under-reporting is a lack of knowledge regarding drug, food, and material allergies.
Horsky et al. (2012)	Systematic review	Review reports describing implementation efforts and collect best available design conventions, procedures and practices in order to provide developers a short compendium of design goals and principles	Developers need to adopt design practices that include user-centered, iterative design and common standards based on human-computer interaction research methods. Specificity and contextual relevance can be

			increased by periodic review of trigger rules, analysis of performance logs and maintenance of accurate allergy, problem and medication lists in health records in order to help avoid excessive alerting.
Hsieh et al. (2004)	Observational retrospective study	Determine characteristics of drug allergy alert overrides, assess how often they lead to preventable adverse drug events (ADEs), and suggest methods for improving the allergy alerting system	80% of the DA alerts were overridden. The most frequent reason for override was "Aware/will monitor" (55%).<br>6% of the overrides resulted in an ADE, but none of them were life-threatening or fatal.<br>Recommendation to only send a trigger for alert when there is an exact match between the ordered drug and allergy list.
Hunteman et al. (2009)	Observational retrospective study	Analyze allergy alerts within a computerized physician order entry system	Of all 643 alerts triggered, 625 (97%) were allergy alerts.<br>Reasons for override were:<br>- medication previously tolerated (49%)<br>- benefit outweighed the risk (29%)<br>- therapeutically appropriate (24%)<br>- other (8%)<br>Allergy information was primarily entered by nurses (70%).
Jani et al. (2011)	Observational retrospective study	Analyze allergy alerts within a computerized physician order entry system	DA conflict alerts were the most accepted type of alert.<br>Prescribers rarely entered an override reason (1.4%), but were most likely to do so for drug-allergy conflicts (95.6%).<br>Reasons for overriding an alert were:<br>- aware / will monitor (68%)<br>- patient tolerates (18%)<br>- patient already taking (14%)
Kesselheim et al. (2011)	Narrative review	Review the principles of product liability to examine whether fears of liability arising from medication-related clinical decision support systems (CDSS) are well grounded, viewed from the standpoint of designers and users	Parsimonious or tailored warnings are overstated, they do not raise the liability risk of system manufacturers and physicians as long as systems are designed well and providers continue to use their best medical judgment.<br>The legal situation surrounding CDSS is unclear. Stronger government regulation could

			help to lower both liability risk and perceptions of risk.
Knight et al. (2015)	Observational retrospective study	Understand factors associated with medication warning acceptance	Of 40,391 warnings, 47% were duplicate warnings, 47% interaction warnings, 6% allergy warnings, and 0.1% adverse reaction warnings.<br>Alert acceptance was positively associated with male patient gender, admission to a service other than internal medicine, caregiver status other than resident, parenteral medications, lower numbers of warnings, and allergy or adverse reaction warning types.<br>Acceptance was more likely when the warning was infrequently encountered and least likely when it was potentially most important.
Kuperman et al. (2003)	Practice experience	Give an overview of the experiences from one large delivery allergy-related decision support system	Organizations should represent allergies in a single location and transportable using communication and content standards.<br>It is important to display warnings that convey to providers the level of importance of the warning.<br>Use strategies such as prompting clinicians when it appears likely that the patient may have had a reaction, or to have patients enter some of their own allergies.
Lee et al. (2014)	Practice experience	Build a real-time adaptive decision support tool to reduce alert fatigue	With the adoption of electronic medical records (EMRs), drug safety alerts are increasingly recognized as valuable tools for reducing adverse drug events and improving patient safety.<br>However, even with proper tuning of the EMR alert parameters, the volume of unfiltered alerts can be overwhelming to users.<br>An adaptive decision support tool in which past cognitive overriding decisions of users are learned, adapted and used for filtering actions, which need to be performed on current alerts to

			increase specificity and reduce the false negative rate.
Lin et al. (2008)	Observational retrospective study	Measure critical order check override rates in VA Puget Sound Health Care System's computerized physician order entry and to compare 2006 with 2001 results	Drug-drug interaction and DA override rates remained high from 2001 to 2006 with significant increases (from 69% to 81%) in drug-allergy order checks. Monitoring override rates must be regular practice in clinical computing systems and qualitative research should be carried out to better understand how physicians interact with decision support at the point of ordering.
Lopez et al. (2012)	Observational retrospective study	Evaluate the use of allergy entries in the electronic medical record (EMR) and to establish the allergy profile of in-patients in a tertiary hospital	Electronic allergy records are a useful tool to identify the allergic background of hospitalized patients and provide information about the allergy or intolerance profile of patients in order to reach optimum quality in the hospital care.<br>Drugs were the most frequent responsible agents (75.63%) followed by foods (11.23%) and materials (4.92%) for having an allergy or intolerance entry. Allergy or intolerance entry was more often used for female patients, compared to males ($P < 0.05$) and proportionally decreased by the age of the patient.
McCoy et al. (2014)	Narrative review	- Elaborate on current trends of evaluation of clinical decision support alerts and their appropriateness<br>- Constitute a proposal to evaluate and improve the appropriateness of alerts in the future	Despite increasing implementation of medication related CDS alerts, detailed evaluations rarely occur. Existing studies solely evaluated alert overrides that are appropriate or justifiable in a single institution. A proposal for improvement should assess alert and response appropriateness, not merely alert overrides. Independent chart review could form a first predictive model that acts as golden standard for the appropriateness. Integrating context and reputation metrics previously showed their use and can also prove their use in the

			medical context.
Nanji et al. (2014)	Observational retrospective study	Characterize the override rates for medication-related clinical decision support (CDS) alerts in the outpatient setting, the reasons cited for overrides at the time of prescribing, and the appropriateness of overrides	About 52.6% medication related CDS alerts of which 16.8% were patient DA alerts, were overridden by providers and 53% of the overrides were classified as appropriate, but the likelihood of overriding an alert varied widely by alert type.<br>Providers cited a variety of reasons for overriding the CDS alerts, with the most common overall being that the patient had previously tolerated the drug. However, the reasons cited for alert overrides varied by alert type.
Russ et al. (2014)	Scenario-based simulation study	Apply human factors engineering principles to improve alert interface design focusing on drug-allergy, drug-drug interaction, and drug-disease alerts	Incorporating human factors principles into alert design significantly improved usability for prescribers and reduced prescribing errors. This study provides some of the first experimental evidence about the presentation of information on computerized medication alerts. This study suggest that a tabular format for presenting multiple alerts and grouping similar information together may aid prescribing decisions. Results indicate that even in an environment where prescribers are likely to shift their cognitive focus from the ordering system to alerts, prescribing errors remained high.
Schiff et al. (1998)	Narrative review	Outline areas in which computer prescribing might transform care processes and patient outcomes	- Choosing drugs should take recommendations as a guideline<br>- Therapy should evolve to a dialogue between patient, provider and computer instead of considering the patient as a passive actor<br>- Screening for drug-drug, drug-allergy and drug-disease interactions requires reliable software and information<br>- Link pharmacy and laboratory information<br>- Prescribing and scheduling of medication go hand-in-hand

			- Medication documentation among care team members should be better coordinated, education can help here<br>- Computers help in facilitating adverse effect documentation<br>- Capturing feedback, e.g. when drug treatment is discontinued, can help in the care process
Shah et al. (2006)	Observational retrospective study	Improve clinician acceptance of drug alerts by designing a selective set of drug alerts for the ambulatory care setting and minimizing workflow disruptions by designating only critical to high-severity alerts to be interruptive to clinician workflow	There were 18,115 drug alerts generated during the study period. Of these, 12,933 (71%) were non-interruptive and 5,182 (29%) interruptive. Of the 5,182 interruptive alerts, 67% were accepted.<br>These data suggest that it is possible to design computerized prescribing decision support with high rates of alert recommendation acceptance by clinicians. This was obtained by using a selective set of clinically significant drug alerts for the ambulatory care setting and minimizing the number of workflow disruptions by designating only critical to high-severity alerts to be interruptive to the clinician workflow.
Slight et al. (2017)	Cross-sectional observational study	Evaluate how often and why providers overrode drug allergy alerts in both the inpatient and outpatient settings	DA alerts are one of the most frequently displayed alerts in computerized physician order entry systems. Most of the DA alerts in this study were overridden, with >94% of a random subsample of alert overrides considered appropriate in both in- and outpatient setting.<br>Reasons for overrides varied dependent on setting, the most prominent were:<br>- patient has taken previously without allergic reaction<br>- physician aware<br>- low-risk cross-sensitivity, will monitor<br>- no reasonable alternative<br>It is very important that the information contained in patients' DA lists is correct and up to date, and clinicians are

			encouraged to deactivate allergies by providing them with an automatic link to update their patients' DA lists.
Stultz et al. (2012)	Systematic review	Analyze the effect of CDSS implementation on medication prescribing and use in pediatrics	Alert-based CCDS functionalities, such as duplicate therapy and medication allergy checking, may generate excessive alerts. Although certain medication-related CCDS functionalities have shown benefit in medication prescribing for pediatric patients, others have resulted in high override rates and inconsistent or unknown impact on patient care. Further studies analyzing the effect of individual CCDS functionalities on safe and effective prescribing and medication use are required.
Swiderski et al. (2007)	Observational retrospective study	Quantify the frequency of allergy warnings that were overridden, specify the reasons why a clinician chose to override an alert, and evaluate allergy warnings that were accepted by prescribers	44% of the warnings resulted in acceptance by the clinician.<br>The primary reason selected for overriding an allergy warning was that the patient tolerated the medication in the past (70%). Of the 342 warnings that were accepted by ordering clinicians, 24% of the warnings were drug specific, and 76% were drug class warnings.<br>Clinicians were much less likely to override a warning if it was an exact drug match compared with a drug class warning. Limiting the number of alerts for less significant drug class warnings would help to improve the positive predictive value of computerized prescriber order entry decision support for allergy warnings.
Tamblyn et al. (2008)	Cluster randomized controlled trial	Test the effectiveness of two approaches to medication alert customization to reduce prevalence of prescribing problems: on-demand versus automatic CDSS	Physicians in the computer-triggered group saw more alerts than the on-demand group, made more changes to the level of alerts they would see, but ignored 87.8% of the problems identified by the computer system.<br>In contrast, on-demand physicians rarely requested drug

			review and thus saw only 1% of the prescribing problems identified by the drug knowledge system, but ignored only 24.4% of problems identified through their requests for advice.<br>In general, both groups underused the drug decision support system. As a result, there was no significant reduction in the overall prevalence of prescribing problems by the end of the follow-up period, except for therapeutic duplication problems in the computer triggered group.
Taylor et al. (2004)	Observational retrospective study	Assess the feasibility and performance of automated drug alerts within an electronic decision support system for physician prescribing	Non-adherence to alert information appeared to be associated with additional knowledge of the clinical situation, beyond that inherent in the decision support tool, for the specific patient context. The most common alerts ignored were: patient known to be intolerant to medication; possible dosing errors; therapeutic duplications; and allergic reactions. Further work is required to understand how to best provide this type of support to physicians.
Topaz et al. (2015)	Observational retrospective study	Explore the common drug allergy alerts over the last 10 years and the reasons why providers overrode these alerts	More than half of the override reasons pointed to irrelevant alerts (i.e. patient has tolerated the medication before, 50.9%) and providers were significantly more likely to override repeated alerts (89.7%) rather than first time alerts (77.4%, $P < 0.001$). There is an urgent need for more efforts to provide more accurate and relevant DA alerts to help reduce alert override rates and improve alert fatigue.
Topaz et al. (2016)	Observational retrospective study	Examine trends in DAI alert overrides for opioid medications	An increasing rate of DAI alert overrides was found, culminating in a 90% rate in this study. Further investigation into providers' reasons for high override rate is necessary. User interfaces and drug-alerting algorithms should evolve to

			enable less interruptive and more accurate alerts to decrease alert fatigue.
Weingart et al. (2009)	Survey	Examine satisfaction of ambulatory clinicians with e-prescribing systems, perceptions on alerts and their perceptions of behavior changes resulting from alerts	E-prescribers were highly satisfied with e-prescribing in general. In contrast, many clinicians perceived alerts to be a nuisance, often triggered by discontinued drugs retained on the medication list or the appropriate use of drug combinations. A novel finding of this study is that alerts also prompted prescribers to take other actions to improve the quality of care, including counseling patients about potential reactions, looking up information in a medical reference, changing the way a patient was monitored, or discussing a case with a colleague.
Weingart et al. (2009)	Focus group study	Understand the reasons for adoption and use of e-prescribing, as well as clinicians' complaints about and perceived benefits of drug allergy and interaction alerts	The decision to adopt e-prescribing was driven largely by financial incentives offered by insurers, and was viewed as a step toward implementation of an electronic medical record. Although participants agreed that the system was easy to learn, few anticipated efficiencies were realized until clinicians configured the device to meet their needs. Electronic prescribing is a potential boon to ambulatory medical practice, although its value may be compromised by inappropriate and irrelevant medication safety alerts and by features of the e-prescribing system that prove burdensome to frontline clinicians. While alerts infrequently result in changed or aborted prescriptions, they may trigger a variety of other provider behaviors that help to ensure safe care.
OUTCOMES			
Bates et al. (1998)	Before and after interventional study and	Evaluate the efficacy of 2 interventions (computerized physician order entry (CPOE) and	Comparing phase 1 (baseline) and phase 2 (after CPOE intervention):

	randomized controlled trial	CPOE in combination with a team intervention) for preventing non-intercepted serious medication errors	- number of serious medication errors decreased significantly from 10.7 events to 4.86 events per 1,000 patient-days (*P* = 0.01)<br>- decline occurred for all stages of the medication use process (from ordering to administering)<br>- preventable ADEs declined from 4.69 events to 3.88 events per 1,000 patient-days (*P* = 0.37)<br>- non-intercepted potential ADEs declined from 5.99 events to 0.98 events per 1,000 patient-days (*P* = 0.002)<br>Comparing within phase 2 CPOE and the combination of CPOE plus a team intervention:<br>- no additional benefit compared to the intervention with only CPOE was seen.
Bates et al. (1999)	Prospective time series analysis	Evaluate the impact of computerized physician order entry (CPOE) with decision support on the number of medication errors	CPOE with clinical decision support decreased the number of medication errors significantly (dose error, frequency error, route error, substitution error and drug-allergy errors). Non-intercepted serious medication errors (those with the potential to cause injury) decreased by 86 % (*P* < 0.001).
Beccaro et al. (2010)	Before and after interventional study with seven sampling periods	Adjust the decision support to reduce the number of alerts (dose checking, DDIs , DAIs)	A team of domain experts worked together to review clinical decision rules.<br>Relative risk(s) (RR) were higher at the start of the intervention versus later for getting: (1) dose range alerts (RR=2.40 [2.28-2.52] *P* < 0.001); and (2) drug-drug interaction alerts (RR=1.63 [1.60-1.66] *P* < 0.001);<br>and was lower at the start of the intervention versus later for getting drug-allergy alerts (RR=0.56 [0.53-0.60] *P* < 0.001).
Benkhaial et al. (2009)	Retrospective cohort study	Compare quality of handwritten allergy information with electronic coding of this information and assess value of International Classification of Diseases, version 10 (ICD-10) codes for drug-allergies as triggers for clinical decision	There was no difference in number of prescribing errors due to drugs potentially inducing allergies when the allergy was only documented as an ICD-10 code or the information was available in the paper record (*P* > 0.05).

		support	This analysis suggests that optimizing the code system and encouraging to document allergies may help in preventing prescribing errors and decreasing the over-alerting rate.
Evans et al. (1998)	Before and after interventional study	Study the use of a computerized decision-support program for the use of antibiotics and other anti-infective agents	The use of the computer-assisted management program for antibiotics and other anti-infective agents led to significant reductions in orders for drugs to which the patients had reported allergies, excess drug dosages, and antibiotic-susceptibility mismatches ($P < 0.01$). There were also marked reductions in the mean number of days of excessive drug dosage ($P < 0.01$) and in adverse events caused by anti-infective agents ($P = 0.02$).
Fung et al. (2003)	Return on investment analysis	Conduct a return on investment analysis of a future CDSS implementation	It is estimated that the addition of decision support will reduce adverse drug events by 4.2 – 8.4%. Based on this estimate, a total net saving of $44,000 - $586,000 is expected over five years. The breakeven period is estimated to be between two to four years.
Harolds et al. (2016)	Narrative review	Investigate the advantages and disadvantages of CPOE systems	CPOE systems linked to CDS have the potential to reduce ADEs, save money, result in better turnaround time for laboratory tests and radiology examinations, increase the adherence of clinicians to following appropriate guidelines, and generally raise the quality and safety of healthcare (correct drug dose, correct drug, ...). Unfortunately, CPOE systems are very time consuming to operate, have too many needless alerts (both in DA checking as in drug-drug interaction findings), do not always reduce the number of significant ADEs as much as was hoped, and introduce new ways to commit errors.
Hsieh et al. (2004)	Observational retrospective study	Determine characteristics of drug allergy alert overrides, assess how often they lead to	80% of the DA alerts were overridden. The most frequent reason for override was

		preventable adverse drug events (ADEs), and suggest methods for improving the allergy alerting system	"Aware/will monitor" (55%).<br>6% of the overrides resulted in an ADE, but none of them were life-threatening or fatal.<br>Recommendation to only send a trigger for alert when there is an exact match between the ordered drug and allergy list.
Kaushal et al. (2003)	Systematic review	Evaluate the effect of CPOE and CDSS on medication safety	5 articles assessed CPOE:<br>- 2 demonstrated a marked decrease in the serious medication error rate;<br>- 1 demonstrated an improvement in corollary orders;<br>- 1 demonstrated an improvement in 5 prescribing behaviors;<br>- 1 demonstrated an improvement in nephrotoxic drug dosing and frequency<br><br>7 articles assessed isolated CDSS:<br>- 3 demonstrated statistically significant improvements in antibiotic-associated medication errors or adverse drug events;<br>- 1 demonstrated an improvement in theophylline-associated medication errors;<br>- 3 studies observed non-significant results.
OUTCOMES			
Kuperman et al. (2001)	Practice experience	Study the impact of CPOE on physician decision making and patient safety	A reduction of serious medication errors by the introduction of CPOE was acknowledged. Also the medication error rate (excl. of dosing) decreased by introducing different levels of decision support (from basic to more advanced clinical decision support (CDS).<br>CPOE and several forms of CDS targeted at increasing patient safety substantially decreased the frequency of serious medication errors and had an even bigger impact on the overall medication error rate.
Leu et al. (2013)	Estimation of financial impact	Implement computer systems for reducing the risk of re-exposure and to evaluate the financial impact of these approaches	About 10% of medication errors relate to drugs given to patients with known allergies; this is translated into between 2,100

		expressed in cost avoidance per year in Taiwan	and 4,200 events of repeated drug allergies per year in Taiwan. Estimated cost avoidance will be as high as NT$ 0.6 million per year (based on the assumption that the cost of repeated DA treatment is about NT$ 13,000) with a range of NT$ 0.1 million to 1.2 million, through a computerized physician order entry in combination with allergy warning and blocking system.
Mahoney et al. (2007)	Before and after interventional study	Implementation of vendor-based integrated clinical information technology was studied, and its effect on medication errors throughout the medication-use process in a health care system was evaluated.	A positive effect of implementation of an integrated clinical information system on medication errors throughout the medication-use process was demonstrated.<br>Most prescribing errors decreased significantly in the selected categories monitored, specifically DA detection (OR=0.14, [95% CI 0.11-0.17]), excessive dosing (OR=0.68, [95% CI 0.62-0.74]), and incomplete or unclear orders (OR=0.35, [95% CI 0.32-0.38]) ($P < 0.001$).
Oliven et al. (2005)	Non-randomized controlled trial	Quantify the impact of CPOE on the incidence and type of prescription errors in the medical service and delineate the causes for remaining errors	CPOE supported in making less significant prescribing errors (PEs): in department A (handwritten) 11.3 errors per 100 hospital days were made compared to department B (provided with CPOE) where only 3.2 errors per 100 hospital days were measured ($P < 0.001$).<br>Only for drug-allergy errors, no significant difference was found.<br>In both departments (A & B), most PEs were caused by the lack or insufficient consideration of abnormal laboratory results.<br>Linking the CPOE with few, specific, laboratory results had a large impact on the prevention of PEs.
Schiff et al. (1998)	Narrative review	Outline areas in which computer prescribing might transform care processes and patient outcomes	- Choosing drugs should take recommendations as a guideline<br>- Therapy should evolve to a dialogue between patient, provider and computer instead of considering the patient as a

			passive actor<br>- Screening for drug-drug, drug-allergy and drug-disease interactions requires reliable software and information<br>- Link pharmacy and laboratory information<br>- Prescribing and scheduling of medication go hand-in-hand<br>- Medication documentation among care team members should be better coordinated, education can help here<br>- Computers help in facilitating adverse effect documentation<br>- Capturing feedback, e.g. when drug treatment is discontinued, can help in the care process
Stultz et al. (2012)	Systematic review	Analyze the effect of CDSS implementation on medication prescribing and use in pediatrics	Alert-based CCDS functionalities, such as duplicate therapy and medication allergy checking, may generate excessive alerts. Although certain medication-related CCDS functionalities have shown benefit in medication prescribing for pediatric patients, others have resulted in high override rates and inconsistent or unknown impact on patient care. Further studies analyzing the effect of individual CCDS functionalities on safe and effective prescribing and medication use are required.
van der Linden et al. (2013)	Systematic review	Identify systems (electronic and non-electronic) that can prevent the represcription of drugs withdrawn because of an ADE and the effects of these systems	Multiple systems have been developed to prevent the represcription of drugs withdrawn because of an ADE, but there is limited evidence that these systems are effective. Of 12 studies that compared pre- and post-intervention periods or wards with and without intervention, only 7 showed a reduction in represcription after an adverse drug event.<br>The 5 non-electronic systems identified, used allergy bracelets or allergy labels on hospital medical records or on drug orders to warn health care providers for represcription.

Varkey et al. (2007)	Observational retrospective study	Evaluate the effect of CPOE on pharmacist-intercepted prescription errors in the outpatient setting	Intercepted prescription errors with computerized prescriptions decreased when compared with handwritten prescriptions (4.9% vs. 7.4%; $P = 0.005$). The most common intercepted prescription error involved the dosage form, followed by quantity dispensed, medication dosage, and DA. The pharmacist plays a critical role in the prevention of these prescription errors.

[a]Allergy documentation was defined as complete if the corresponding reaction field contained useful information to provide clinical decision support on whether a drug could be readministered to a patient or not.
