# Supplementary material for: Clinical Decision Support Systems for Drug Allergy Checking: Systematic Review
Source: J Med Internet Res. 2018 Sep 7;20(9):e258. doi: 10.2196/jmir.8206 (PMC6231757; doi:10.2196/jmir.8206)
Supplement: Multimedia Appendix 3 [file jmir_v20i9e258_app3.pdf]

Multimedia Appendix 3. Table with the number of subjects included in the review.

Authors (year)	Number of subjects
DOCUMENTING THE PRESENCE/ABSENCE OF AN ALLERGY	
Burrell et al. (2013) [32]	Patients:<br>- total: 2,174 (pre: 1,016 & post: 1,158)<br><br>Allergies:<br>- total: 1,686 (pre: 770 & post: 916)
Cresswell et al. (2008) [35]	-
Fernando et al. (2014) [30]	Interviews: 21
Ferner et al. (2010) [36]	-
Gay et al. (2009) [33]	Medical and surgical inpatients: 117
Hsieh et al. (2004) [37]	Patients: 1,150 - subset of 320<br>Alerts: 7,761<br>Overrides: 6,182
Kuperman et al. (2007) [14]	-
Lopez-Gonzalez et al. (2009) [38]	Articles: 1630<br>Inclusion: 45
Mawby (2006) [34]	-
Mills (1964) [31]	Member hospitals mailed: 450<br>Responses: 332 (73.7%)
Porter et al. (2006) [39]	Parent-child dyads observed at triage: 256
Rimawi et al. (2013) [40]	Patients with proven penicillin tolerance rehospitalized within a one-year period from the penicillin skin test: 55
Ross et al. (2013) [41]	Prescribers: 40<br>Prescribing errors questioned about: 100
Schiff et al. (1998) [42]	-
CODING	
Abookire et al. (2000) [43]	-
Benkhaial et al. (2009) [44]	Inpatients: 200
Bernstein (2014) [45]	-
Chaffee et al. (2010) [46]	-
Demner-Fushman et al. (2009) [47]	-
Goss et al. (2013) [48]	Terminologies compared: 5
Greibe (2013) [49]	-
Ogallo et al. (2015) [50]	-
Paul et al. (2012) [51]	-
Slight et al. (2015) [52]	Professionals (academicians, practitioners, policy makers and senior management officials): 35
RULE BASES	
Baysari et al. (2013) [53]	Prescribers: 68
Chaffee et al. (2010) [46]	-
Evans et al. (1998) [54]	Patients:<br>- 1,136 (pre-intervention period)<br>- 545 (post-intervention period)
Kesselheim et al. (2011) [55]	-
Kuperman et al. (2007) [14]	-
Mahoney et al. (2007) [56]	Inpatient medication orders:<br>- 1,452,346 (pre-implementation period)<br>- 1,390,789 (post-implementation period)
ALERTS AND ALERT FATIGUE	
Abookire et al. (2000) [43]	-
Ariosto (2014) [57]	Patients: 30,321
Baysari et al. (2013) [53]	Prescribers: 68

Brodowy et al. (2016) [58]	DA alerts per order:<br>- period 1: 0.14<br>- period 2: 0.09
Bryant et al. (2014) [59]	Physicians: 461<br>Alerts: 2,455 / 18,354 orders
Carspecken et al. (2013) [60]	Patients: 1
Chaffee et al. (2010) [46]	-
Coleman et al. (2013) [18]	-
Dekarske et al. (2015) [61]	Clinicians: 22 (2 cohorts)<br>Alerts: 1829<br>- phase 1: 820 (44.8%)<br>- phase 2: 1,009 (55.2%)
Falade et al. (2012) [62]	Patients: 763<br>Medication warnings: 8,235
Genco et al. (2016) [63]	Patient visits: 4,581
González-Gregori et al. (2012) [64]	Patients: 15,534
Horsky et al. (2012) [65]	Articles included: 112
Hsieh et al. (2004) [37]	Patients: 1,150 - subset of 320<br>Alerts: 7,761<br>Overrides: 6,182
Hunteman et al. (2009) [66]	Allergy alerts: 643 / 49,887 (1.3% of total number of all types of alerts)
Jani et al. (2011) [67]	Order items: 26,836<br>Alerts: 16,182<br>- Visible alerts: 3,507<br>- Overridden alerts: 3,119
Kesselheim et al. (2011) [55]	-
Knight et al. (2015) [68]	Patients: 6,646<br>Warnings: 40,391
Kuperman et al. (2003) [69]	-
Lee et al. (2014) [70]	Alerts: 107,917<br>- dosing: 74,312 (68.9 %)<br>- DA: 14,943 (13.8 %)<br>- drug-drug: 18,662 (17.3 %)
Lin et al. (2008) [71]	2001<br>Critical orders: 215/ 42,621 (0.5%)<br>DA order check override: 72 / 105 (69%)<br><br>2006<br>Critical orders: 908 / 37,040 (2.5%)<br>DA order check override: 341 / 420 (81%)
Lopez et al. (2012) [72]	Patients: 15,534<br>EMR of patients containing allergy information: 10,013 (64.45%)
McCoy et al. (2014) [73]	-
Nanji et al. (2014) [74]	Medication orders: 2,004,069<br>CDS alerts: 157,483<br>Alert overrides: 82,889 (52.6%)
Russ et al. (2014) [75]	Prescribers: 40<br>Prescribing errors questioned about: 100
Schiff et al. (1998) [42]	-
Shah et al. (2006) [76]	Drug alerts: 18,115<br>- Noninterruptive: 12,933 (71%)<br>- Interruptive: 5,182 (29%)
Slight et al. (2017) [1]	Patients: 29,420 patients<br>DA alerts: 158,023<br>DA alert override: 128,157 (81%)
Stultz et al. (2012) [19]	Articles included: 44

Swiderski et al. (2007) [77]	Orders: 29,483<br>Allergy alerts: 777<br>- accepted: 342 (44%)<br>- overridden: 435 (56%)
Tamblyn et al. (2008) [78]	Physicians: 28<br>- on-demand CDDS: 14 (50 %)<br>- automatic CDDS: 14 (50 %)<br><br>Patients: 3,449<br>- on-demand CDDS: 1,550 (44.9 %)<br>- automatic CDDS: 1,899 (55.1 %)
Taylor et al. (2004) [79]	Electronic prescriptions: 6,260<br>Drug alerts: 1,869
Topaz et al. (2015) [17]	DA alerts: 611,192
Topaz et al. (2016) [80]	Opioid drug allergy interaction (DAI) alerts: 342,338
Weingart et al. (2009) [81]	Ambulatory care clinicians: 300<br>Responses: 184 (61%)
Weingart et al. (2009) [82]	Clinicians: 25
OUTCOMES	
Bates et al. (1998) [83]	Medical units:<br>- 6 (phase 1) & 8 (phase 2)<br>Admissions:<br>- phase 1: 2,491 & phase 2: 4,220<br>Patient-days:<br>- phase 1: 12,218 & phase 2: 24,539
Bates et al. (1999) [84]	-
Beccaro et al. (2010) [85]	-
Benkhaial et al. (2009) [44]	Inpatients: 200
Evans et al. (1998) [54]	Patients:<br>- 1,136 (pre-intervention period) & 545 (post-intervention period)
Fung et al. (2003) [86]	Physicians: 4,200<br>Allied health professionals: 4,000
Harolds et al. (2016) [87]	-
Hsieh et al. (2004) [37]	Patients: 1,150 - subset of 320<br>Alerts: 7,761<br>Overrides: 6,182
Kaushal et al. (2003) [22]	Articles included: 12
Kuperman et al. (2001) [16]	-
Leu et al. (2013) [88]	-
Mahoney et al. (2007) [56]	Inpatient medication orders:<br>- 1,452,346 (pre-implementation period)<br>- 1,390,789 (post-implementation period)
Oliven et al. (2005) [89]	2 Departments:<br>- A [handwritten] & B [CPOE]<br>Hospitalization days:<br>- 10,002 (A:4,969 & B:5,033)<br>Patients:<br>- 1,350 (A: 641 & B:709)
Schiff et al. (1998) [42]	-
Stultz et al. (2012) [19]	Articles included: 44
van der Linden et al. (2013) [90]	Articles included: 45
Varkey et al. (2007) [91]	Orders: 4,527
